# The Role of Metal Adatoms in a Surface‐Assisted Cyclodehydrogenation Reaction on a Gold Surface

**DOI:** 10.1002/anie.202212354

**Published:** 2022-11-08

**Authors:** Jonas Björk, Carlos Sánchez‐Sánchez, Qiang Chen, Carlo A. Pignedoli, Johanna Rosen, Pascal Ruffieux, Xinliang Feng, Akimitsu Narita, Klaus Müllen, Roman Fasel

**Affiliations:** ^1^ Department of Physics Chemistry and Biology, IFM Linköping University 58183 Linköping Sweden; ^2^ nanotech@surfaces Laboratory Empa, Swiss Federal Laboratories for Materials Science and Technology Überlandstrasse 129 8600 Dübendorf Switzerland; ^3^ ESISNA group Materials Science Factory Institute of Material Science of Madrid (ICMM–CSIC) Sor Juana Inés de la Cruz 3 28049 Madrid Spain; ^4^ Max Planck Institute for Polymer Research Ackermannweg 10 55128 Mainz Germany; ^5^ Current address: Department of Chemistry University of Oxford Chemistry Research Laboratory Oxford OX1 3TA UK; ^6^ Faculty of Chemistry and Food Chemistry & Center for Advancing Electronics Dresden Technische Universität Dresden 01062 Dresden Germany; ^7^ Organic and Carbon Nanomaterials Unit Okinawa Institute of Science and Technology Graduate University 1919-1 Tancha, Onna-son, Kunigami-gun Okinawa 904-0495 Japan; ^8^ Department of Chemistry Biochemistry and Pharmaceutical Sciences University of Bern Freiestrasse 3 3012 Bern Switzerland

**Keywords:** Cyclodehydrogenation, Density Functional Theory, Reaction Mechanisms, Scanning Probe Microscopy, On-Surface Synthesis

## Abstract

Dehydrogenation reactions are key steps in many metal‐catalyzed chemical processes and in the on‐surface synthesis of atomically precise nanomaterials. The principal role of the metal substrate in these reactions is undisputed, but the role of metal adatoms remains, to a large extent, unanswered, particularly on gold substrates. Here, we discuss their importance by studying the surface‐assisted cyclodehydrogenation on Au(111) as an ideal model case. We choose a polymer theoretically predicted to give one of two cyclization products depending on the presence or absence of gold adatoms. Scanning probe microscopy experiments observe only the product associated with adatoms. We challenge the prevalent understanding of surface‐assisted cyclodehydrogenation, unveiling the catalytic role of adatoms and their effect on regioselectivity. The study adds new perspectives to the understanding of metal catalysis and the design of on‐surface synthesis protocols for novel carbon nanomaterials.

## Graphical abstract

Using scanning tunneling microscopy and electronic structure theory, we show that, contrary to previous consensus, surface adatoms can play a central role in the highly chemoselective cyclodehydrogenation of organic polymers.

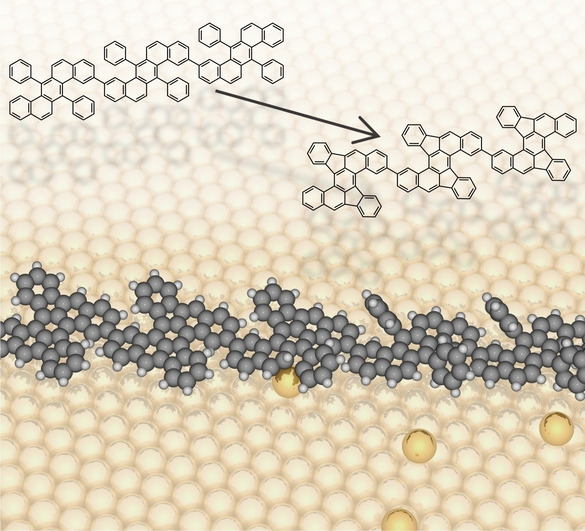

## Introduction

Dehydrogenation reactions—the detachment of hydrogen atoms from a molecule—are at the core of key chemical disciplines, such as organic synthesis or petrochemical refining. These include relatively simple reactions, like styrene formation from ethylbenzene^[1]^ or aromatization of cyclohexane,^[2]^ but also complex biochemical processes, like key steps of the Krebs cycle.^[3]^ In organic synthesis, methods of carbon‐carbon (C−C) bond closure are often accompanied or followed by carbon‐hydrogen (C−H) bond cleavage. Examples of the latter are light‐induced stilbene‐dihydrophenanthrene transformation,^[4]^ which after removal of hydrogen atoms becomes a “photodehydrocyclization”, or the Scholl reaction.^[5]^ There again, C−C bond formation by an electrocyclic step is followed by loss of hydrogen atoms, thus yielding a “cyclodehydrogenation”. Common to the majority of such transformations is the necessity of a stoichiometric “oxidation” reagent or a catalyst—acid, enzymes, or metal. Despite their unarguable importance and strong efforts devoted to the elucidation of the underlying reaction mechanisms, there are still open questions regarding, for example, the mechanisms of heterogeneous catalysis,^[6]^ given the difficulty of obtaining information at the atomic level. In this regard, the advent of *surface science* and, more specifically, of *on‐surface synthesis (OSS)*—a combination of heterogeneous catalysis and organic synthesis^[7]^—has allowed the study of such processes at the atomic level. Thanks to the highly controlled environmental conditions provided by ultra‐high vacuum (UHV), the use of well‐defined single‐crystal surfaces and the application of powerful characterization techniques, unprecedented insights can be obtained at the molecular and atomic scale.^[8]^ Furthermore, these well‐defined conditions are ideal for the theoretical modelling of the reaction mechanisms taking place at interfaces.^[9]^ Additionally, OSS has provided an efficient and elegant route toward the bottom‐up synthesis of unprecedented nanomaterials—many of them not available by conventional wet chemistry because products are often too reactive or not soluble—via inter‐ and/or intramolecular reactions of specifically functionalized organic molecules on (usually metallic) surfaces.

During the last decade, OSS has revealed a plethora of reactions in which dehydrogenation plays a vital role, such as polymerization of alkanes,^[10]^ homo‐coupling of terminal alkynes,^[11]^ as well as various cyclization reactions.[^12^, ^13^] Cyclodehydrogenation (CDH) reactions have been of notable interest for synthesizing low‐dimensional nanostructures such as fullerenes,^[14]^ nanodomes,[^15^, ^16^] graphene and its derivatives,[^17^, ^18^, ^19^, ^20^, ^21^, ^22^] or hexagonal boron nitride.^[23]^ Of particular interest is the bottom‐up synthesis of atomically precise graphene nanoribbons (GNRs),^[24]^ where typically two reaction steps—Ullmann‐like coupling and CDH—take place in a sequential way. The relevance of CDH reactions for the production of materials of (potential) technological relevance makes it highly important to govern these reactions with high precision. Further, given the ground‐breaking role of surface‐assisted dehydrogenation, a comprehensive understanding of the mechanisms of surface‐assisted dehydrogenation is required, and CDH reactions are ideal model cases.

A major question within heterogeneous catalysis in general, and OSS in particular, is the role of metal adatoms.^[25]^ Reactions have mostly been considered to proceed on atomically flat terraces^[26]^ or at step edges.^[27]^ At finite temperatures, however, metal atoms may be released from step edges, populating the terraces by a 2D gas of metal adatoms.[^28^, ^29^] Such adatoms are often responsible for the formation of metal‐coordinated networks on copper and silver surfaces.[^30^, ^31^, ^32^] Furthermore, theoretical modelling has shown that freely available adatoms have the potential of lowering the activation energies of dehydrogenation on both Cu(111)^[33]^ and Au(111).^[34]^ However, whether adatoms actually take part in dehydrogenation reactions relies on their availability. In other words, the chemical potential of the adatoms determines whether they lower the free energy of activation or not. To the best of our knowledge, there is no experimental identification of adatoms that activate CDH reactions on surfaces so far.

**Figure 1:**
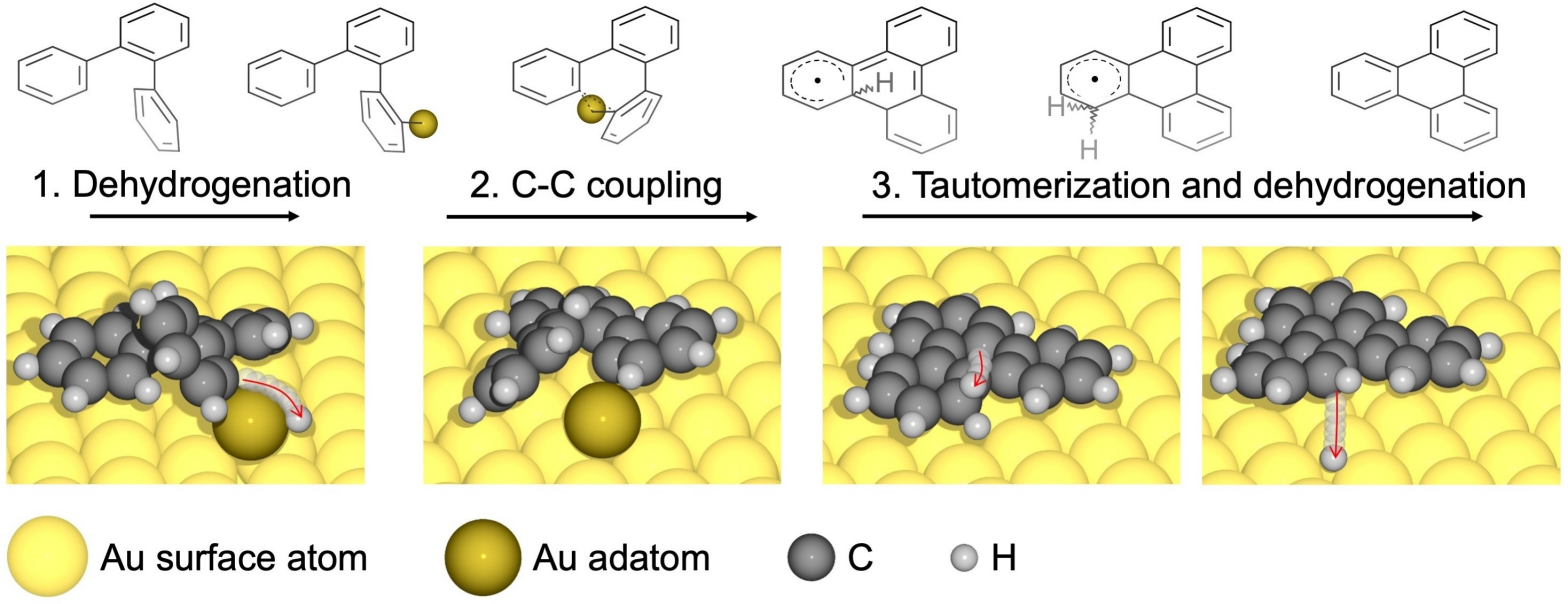
Illustration of the mechanism of adatom‐mediated cyclodehydrogenation on Au(111). Dehydrogenation is induced by a thermally generated adatom, followed by adatom‐mediated C−C bond formation. The cyclodehydrogenation is finalized by a tautomerization step and subsequent dehydrogenation for which the adatom does not participate. In the dehydrogenation and tautomerization steps, the paths of the involved hydrogen atoms are indicated by red arrow. The colors of the different atom types are indicated at the bottom of the figure.

**Figure 2:**
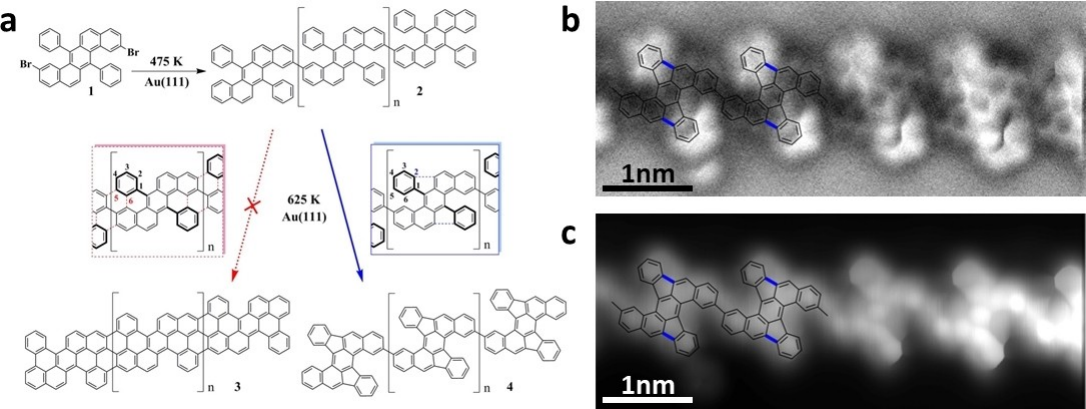
a) Schematics of the polymerization of 2,9‐dibromo‐7,14‐diphenylbenzo[*k*]tetraphene (**1**) into polymer **2**. Following the formation of polymer **2**, two CDH pathways involving the phenyl substituents are possible: they may either form two six‐membered rings resulting in a cove‐edged graphene nanoribbon (**3**, red path), or a five‐membered ring yielding a new type of polymer consisting of dibenzo[*a*,*m*]rubicene repeat units (**4**, blue path). The nc‐AFM frequency‐shift image (b) and simultaneously recorded current image (c) clearly reveal the formation of five‐membered rings, and thus identify **4** as the experimentally obtained reaction product (*V*=−5 mV).

Here, we provide chemical insight into the CDH reaction sequence by means of scanning tunnelling and non‐contact atomic force microscopies (STM & nc‐AFM), and density functional theory‐based transition state theory calculations (DFT‐TST). Specifically, and different from the prevalent understanding of the mechanism of surface‐assisted CDH, we propose that the CDH is triggered by dehydrogenation prior to C−C bond formation, with the former being driven by thermally generated adatoms on the Au(111) surface, as illustrated in Figure 1. The corresponding reaction pathway is characterized by a sequence of three steps: (1) dehydrogenation, (2) C−C bond formation, and (3) tautomerization(s) with subsequent dehydrogenation. Steps (1) and (2) are assisted by thermally generated Au adatoms, in contrast to the current consensus that the reaction would be initiated by C−C bond formation on the flat surface. These insights are obtained by utilizing a 2,9‐dibromo‐7,14‐diphenylbenzo[*k*]tetraphene as the molecular precursor (**1**, see Figure 2). Upon adsorption and gentle thermal activation, **1** transforms into polymer **2**. In the absence or presence of adatoms, our calculations predict that the CDH of precursor **2** provides graphene nanoribbon **3** or polymer **4**, respectively. Importantly, only product **4** is observed experimentally, which supports the adatom‐activated pathway. These results open new perspectives on the on‐surface CDH reaction mechanisms and highlight the hitherto largely overlooked role of adatoms in surface reactions.

## Results and Discussion

When deposited onto a clean Au(111) surface held at 475 K, monomer **1** (see the Supporting Information for details on monomer synthesis and characterization) undergoes surface‐assisted Ullmann‐like coupling into polymer **2** (see Figure S1 and S2 for further details on the polymerization step). Considering polymer **2** as the starting point of the CDH reaction, two possibilities arise: the formation of two additional six‐membered rings via two new C−C bonds at C atoms denoted as **C5** and **C6** in Figure 2a (red box), or the formation of a five‐membered ring at **C2** position (blue box). The former sequence would yield the cove‐edged chiral GNR **3**, while the latter would result in polymer **4**. Figure 2b shows a representative frequency‐shift nc‐AFM image after the CDH reaction at 625 K, in which the non‐planar geometry of **4** is evidenced, as is expected upon the formation of the five‐membered rings. Additional information is provided by the simultaneously recorded current image in Figure 2c, in which the internal structure of **4** is resolved. Effectively, **4** consists of a benzo[*k*]tetraphene backbone flanked by one indeno unit on each side, forming a dibenzo[*a*,*m*]rubicene structure, corroborating the formation of polymer **4**. Further details on the STM and nc‐AFM analysis, including images at different tip‐sample distances, are provided in the Supporting Information (Figures S3 and S4). Note that these polymers are rather long (see Figure S5), probably due to the higher flexibility of the monomer precursor that enables efficient polymerization in the first stage, and exclusively formed by non‐benzenoid dibenzo[*a*,*m*]rubicene molecular units (see Figure S6).

To rationalize the preferred formation of five‐ over six‐membered rings and to elucidate the underlying reaction mechanism, we performed DFT‐TST calculations. As discussed below, only the presence of adatoms can explain the observed reaction pathway, which we substantiate by comparing a range of plausible reaction paths. See Supporting Information for a detailed account of all calculations, for which both calculated enthalpies and free energies (including the corresponding entropy of the associative desorption of a H_2_ after each dehydrogenation step) are indicated for all considered pathways. When comparing different reactions, we refer to the free energies.

There are two fundamental ways for the CDH reaction to be initiated on a surface: by C−C bond formation or by dehydrogenation. On the atomically flat surface, i.e. without metal adatoms, our theoretical results indicate that the reaction is more efficiently initiated by dehydrogenation, with a difference in activation energy of about 0.1 eV (1.85 eV for C−C coupling and 1.76 eV for dehydrogenation, Figure S7–S9). Such a small energy difference indicates that dehydrogenation and C−C coupling are competing processes on the flat surface at the reaction temperature of 625 K, with a slight preference for dehydrogenation. However, the dehydrogenation on the flat surface is predicted to result in mainly six‐membered and only a small number of five‐numbered rings (Figure S8 and S9) as it will be discussed in detail below, contradicting the experimental observations.

**Figure 3:**
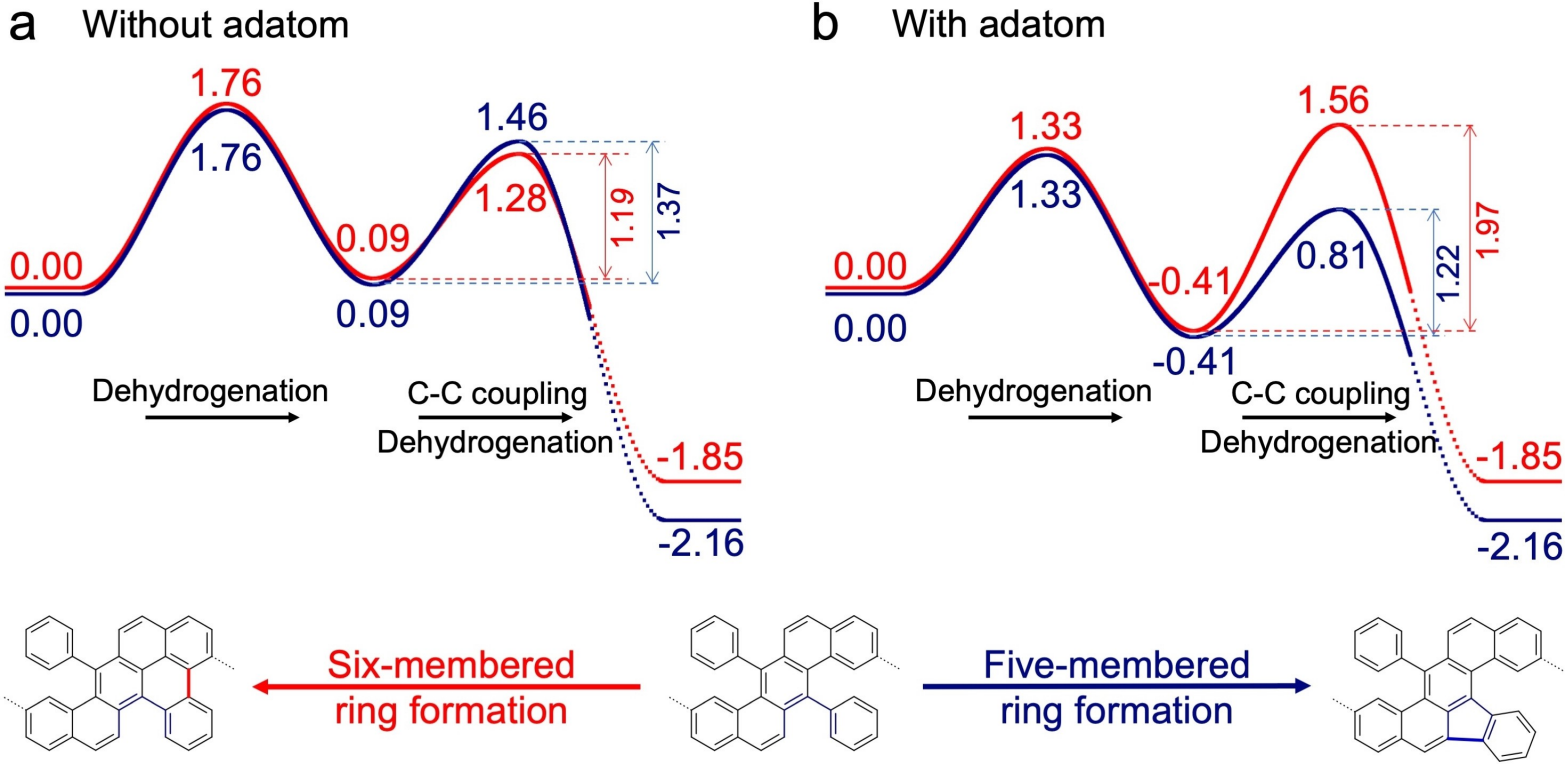
Comparison between reactions without adatom (a) and with adatom (b) for the formation of a six‐ (red curves) and a five‐membered ring (blue curves). Only the presence of an adatom in the initial dehydrogenation step can explain the preference towards formation of five‐membered rings. For each reaction, the C−C bond formation is accompanied by a dehydrogenation step, but only the barrier for the former is shown, while the final energy corresponds to the energy of the fully cyclodehydrogenated products. The entire CDH sequence of these multistep processes can be found in the Supporting Information (Figure S8 and S9 without and Figure S10 and S11 with adatoms). Units in eV.

Instead, Au adatoms appear to be crucial for explaining the observed formation of exclusively five‐membered rings. This becomes clear when comparing the formation of a six‐ and a five‐membered ring following the initial dehydrogenation. The barriers of dehydrogenation and subsequent C−C bond formation, computed both by including or not including an adatom, are depicted in Figure 3. In each case, the C−C coupling implies the release of a second hydrogen atom through a multistep pathway (see Supporting Information for details about the reaction pathways). Without adatom, the barrier to form a six‐membered ring (red curve) is slightly lower than that of a five‐membered ring (blue curve). Although the difference is relatively small (0.18 eV), this should lead to the preferential formation of six‐membered rings, and at most a slight admixture with five‐membered rings. This is in clear contradiction with experiments, where five‐membered rings are formed exclusively. Consideration of the corresponding reaction pathways assisted by an adatom convincingly rationalizes the observed behavior (Figure 3b). Following initial dehydrogenation, the formation of a six‐ and a five‐membered ring has activation energies of 1.97 eV and 1.22 eV, respectively. This corresponds to a $2\times 10^{6}$ times larger Boltzmann factor for the latter process at 600 K, which renders the formation of the six‐membered ring highly unlikely and, thus, leads exclusively to five‐membered rings. This is in accordance with experimental observations and provides strong support for adatoms catalyzing surface‐assisted CDH. Note that the main effect of the adatom for the C−C coupling is that it increases the activation energy for the six‐membered ring formation (from 1.19 eV to 1.97 eV), while it slightly decreases that of the five‐membered ring formation (from 1.37 eV to 1.22 eV).

Simply comparing the activation of the initial dehydrogenation with and without adatom is, however, not sufficient to conclude that the CDH is driven by adatoms as the calculated activation energy with adatom depends on the adatom's chemical potential. The conclusion is rather based on the fact that only five‐membered rings are formed in the experiments, and it is only the adatom‐driven pathway that corroborates the preference of this reaction product. One may hypothesize that other irregularities of the surface, such as step edges, are important catalytic centers for the reaction. The case of step edges can be disregarded based on the experimental data. Since all polymers cannot easily access a step edge, we would see a strong dependence on surface coverage if they were driving the reaction. In any case, we also considered a scenario where the initial dehydrogenation is initiated by a group of three adatoms, instead of a single adatom. However, in such a scenario, the barrier is 1.72 eV (Figure S12 of the Supporting Information), i.e., comparable to that without adatom. It is also of interest to understand under which conditions the adatom‐assisted dehydrogenation can be anticipated to dominate over the dehydrogenation without adatom. In the Supporting Information (Figure S14 and corresponding text), we discuss this in terms of the entropy difference between a free and bonded Au adatom, providing a rough estimate for the conditions under which adatoms become important. We would like to stress, however, that our conclusion that CDH is driven by adatoms is based on the strong preference of five‐membered ring formation, which is only explained by the participation of adatoms.

**Figure 4:**
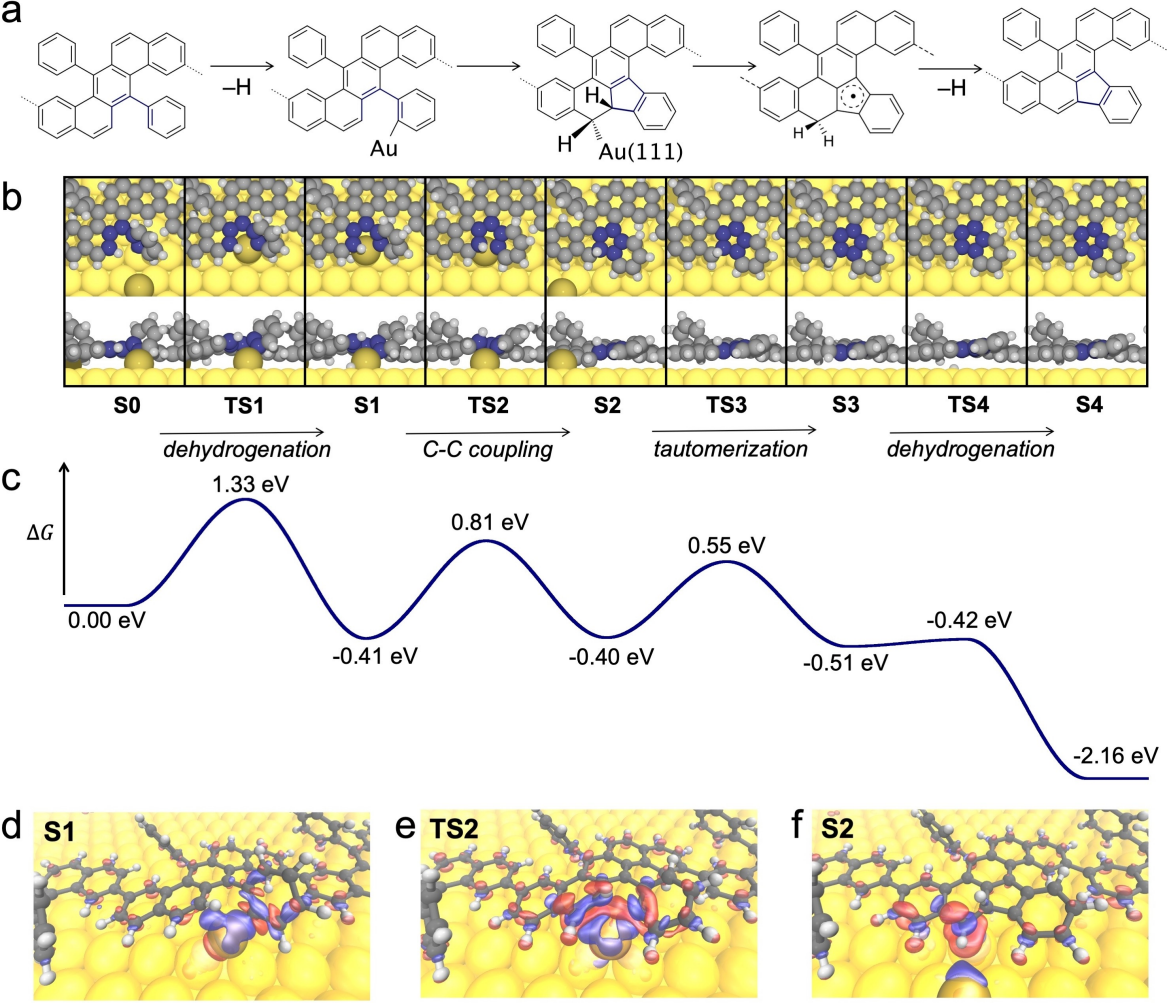
a–c) Reaction mechanism for the formation of a single pentagon with a) valence bond structures of local minima (**S0**–**S4**) together with b) top and side views of both local minima and transition states (**TS1**–**TS4**) and c) corresponding free energy profile including entropy of associatively desorbed H_2_ after each dehydrogenation step. d–f) Surface plots of electron density differences with blue (red) corresponding to electron accumulation (depletion).

To understand the precise role of the adatom, the complete lowest‐energy reaction pathway is considered in Figure 4. In the dehydrogenation step (**S0**–**S1**), the gold adatom activates the C−H bond by interacting with both the carbon and the hydrogen atoms and inserting to the C−H bond (the abstracted hydrogen is obscured by the adatom and molecule in **S0** and **TS1**). The dehydrogenation includes the release of the hydrogen from the adatom and the associative desorption of H_2_ (see below). The origin of the lower energy barrier towards five‐membered ring formation can be understood by considering the bonding configuration of the Au adatom following the formation of this organometallic species (**S1**). The phenyl group with the Au adatom is naturally tilted toward the subsequent formation of the five‐membered ring, while there is an additional energy cost to rotate the phenyl group and Au adatom over into the configuration suitable for C−C coupling into the six‐membered ring (see Figure S10 and S11 for a comparison between the formation of five‐ and six‐membered rings). The adatom is released from the molecule simultaneously with the C−C bond formation (**S1**–**S2**), reminiscent of reductive elimination. The entire process is then finalized by subsequent tautomerization (**S2**–**S3**) and dehydrogenation (**S3**–**S4**). The rate‐limiting step of the overall process is the initial insertion of the metal adatom to the C−H bond—all other steps occur at shorter timescales (e.g., the Boltzmann factor of crossing **TS2** from **S1** is about ten times larger than crossing **TS1** from **S0** at 600 K). It should be noted that the overall reaction is endothermic (+0.21 eV) and that the gain in free energy, making the reaction exergonic, originates from the abstracted hydrogen desorbing associatively as H_2_ from the surface, with each hydrogen molecule contributing with −2.37 eV at the temperature of 600 K and pressure of 10^−12^ bar assumed in our calculations. The desorption of hydrogen, either associatively as H_2_ or as HBr when surface‐adsorbed Br is available (as byproduct of the polymerization step), is a well‐known phenomenon in on‐surface synthesis^[35]^ and thermodynamically possible due to the entropy gain of this process.^[36]^

Our calculations, thus, attribute the reason for the exclusive formation of five‐membered rings to the role of Au adatoms. This behavior refutes any skepticism against sufficient presence of adatoms to drive the reaction forward. Indeed, on close‐packed metal surfaces, adatoms are known to appear and diffuse across terraces at elevated temperatures, where they continuously detach from step edges.[^28^, ^37^]

Figure 4 also provides additional information on the function of the Au adatom in the reaction, as reflected by electron density difference plots for the intermediates **S1** and **S2** and the transition state **TS2** (Figure 4e–f, respectively). In the first intermediate state (**S1**), a chemical bond has been established between the adatom and the dehydrogenated C atom, which is reflected by the electron accumulation between the two atoms (blue cloud). Furthermore, there is a slight accumulation of electron density between the Au adatom and the C atom that later contributes to the formation of the new C−C bond, closing the five‐membered ring. In other words, following the initial dehydrogenation, the Au adatom forms a weak organo‐metallic bridge between the two C atoms participating in the coupling. In the transition state of the C−C bond formation (**TS2**, Figure 4e), the chemical role of that adatom becomes even more obvious as manifested by the strong charge redistribution between the adatom and the C atoms taking part in the coupling (as well as adjacent C atoms). Thus, the Au adatom is not only important for promoting the dehydrogenation, but also facilitates the C−C bond formation step. Finally, in the intermediate state following the C−C bond formation (**S2**, Figure 4f), the Au adatom disconnects from the molecular system, while a chemical bond is established between a neighboring C atom and the surface.

Despite the strong influence of adatoms shown by our calculations, it remains to be seen how general this trend is, i.e., whether one can expect adatom‐assisted CDH (initiated by dehydrogenation) to be a general phenomenon or whether it is specific to the present system. To test the generality of the reaction pathway, we have also calculated different pathways of the simplest relevant CDH reaction conceivable on Au(111), namely the conversion of *ortho*‐terphenyl into triphenylene (Figure 1; Supporting Information, Figure S15–S18). Table 1 lists the activation energies for the different mechanisms of initiating the reaction (direct C−C coupling and dehydrogenation, without and with adatom) and compares these values to the corresponding activation energies relevant for our title polymer **2**. What one observes is a striking resemblance of the activation energies for the respective processes in the two systems. In the absence of adatoms, dehydrogenation is slightly more likely than direct C−C bond formation. Most importantly, the activation energy is reduced by about 0.4 eV in both systems when being assisted by a gold adatom. This substantiates the pivotal role that adatoms can play in CDH reactions on Au(111), although further studies will be needed to fully generalize this statement. It should be noted that, of course, the subsequent steps following initial dehydrogenation depend on the system of choice and govern the final structure.

**Table 1:** Comparison of the activation energies for different ways of initiating the CDH for *ortho*‐terphenyl and polymer **2**, as obtained from our calculations. In the case of polymer **2**, the direct C−C coupling refers to the formation of a five‐membered ring, which has lower barrier than the simultaneous formation of two C−C bonds resulting in a six‐membered ring. The details of all pathways are given in the Supporting Information.

	*Ortho*‐terphenyl	Polymer **2**
Direct C−C coupling (without adatom)	1.88 eV	1.85 eV
C−H activation (without adatom)	1.78 eV	1.76 eV
C−H activation (with adatom)	1.37 eV	1.33 eV

## Conclusion

We have presented novel perspectives on the mechanism of CDH reactions on the Au(111) surface. Unlike the prevalent understanding, in which CDH is initiated by C−C bond formation and followed by hydrogen transfer reactions, we have shown that on‐surface CDH can be triggered by dehydrogenation catalyzed by thermally generated Au adatoms. This profound insight was obtained by studying a polymer that may form either six‐ or five‐membered rings through CDH, while only the latter are observed experimentally. DFT‐TST calculations suggested that the observed reaction behavior was possible only by the presence of adatoms, driving the initial dehydrogenation and subsequent C−C bond formation of the overall reaction. To challenge the generality of the mechanism, we calculated the CDH of a simple model compound, which confirmed the proposed reaction sequence. As exemplified by our study, the detailed mechanism of CDH is decisive for the atomistic structure of a formed material and a correct fundamental understanding of a reaction is paramount to devise synthesis protocols for targeted nanostructures. Our results are anticipated to substantially contribute to the quest for novel carbon‐based nanomaterials from rationally designed molecular precursors reacting in a pre‐programmed way when deposited on a surface.

## Conflict of interest

The authors declare no conflict of interest.

1

## Supporting information

As a service to our authors and readers, this journal provides supporting information supplied by the authors. Such materials are peer reviewed and may be re‐organized for online delivery, but are not copy‐edited or typeset. Technical support issues arising from supporting information (other than missing files) should be addressed to the authors.

Supporting Information

## Data Availability

The data that support the findings of this study are available from the corresponding author upon reasonable request.

## References

[1] D. H. James , W. M. Castor , in Ullmann's Encycl. Ind. Chem., Wiley-VCH, Weinheim, 2011, pp. 529–544.

[2] M. C. Tsai , C. M. Friend , E. L. Muetterties , J. Am. Chem. Soc. 1982, 104, 2539–2543.

[3] H. A. Krebs , W. A. Johnson , Biochem. J. 1937, 31, 645–660. 16746382 10.1042/bj0310645 PMC1266984

[4] F. B. Mallory , C. W. Mallory , in Org. React. 1984, pp. 1–456.

[5] B. T. King , J. Kroulík , C. R. Robertson , P. Rempala , C. L. Hilton , J. D. Korinek , L. M. Gortari , J. Org. Chem. 2007, 72, 2279–2288. 17326684 10.1021/jo061515x

[6] K. F. Kalz , R. Kraehnert , M. Dvoyashkin , R. Dittmeyer , R. Gläser , U. Krewer , K. Reuter , J. D. Grunwaldt , ChemCatChem 2017, 9, 17–29. 28239429 10.1002/cctc.201600996 PMC5299475

[7] A. Gourdon , Angew. Chem. Int. Ed. 2008, 47, 6950–6953; 10.1002/anie.200802229 18683834

[8] S. Clair , D. G. De Oteyza , Chem. Rev. 2019, 119, 4717–4776. 30875199 10.1021/acs.chemrev.8b00601 PMC6477809

[9] J. Björk , J. Phys. Condens. Matter 2016, 28, 083002–083016. 26836411 10.1088/0953-8984/28/8/083002

[10] D. Zhong , J. H. Franke , S. K. Podiyanachari , T. Blömker , H. Zhang , G. Kehr , G. Erker , H. Fuchs , L. Chi , Science 2011, 334, 213–216. 21998384 10.1126/science.1211836

[11] Y.-Q. Zhang , N. Kepčija , M. Kleinschrodt , K. Diller , S. Fischer , A. C. Papageorgiou , F. Allegretti , J. Björk , S. Klyatskaya , F. Klappenberger , M. Ruben , J. V. Barth , Nat. Commun. 2012, 3, 1286. 23250416 10.1038/ncomms2291

[12] A. Riss , A. P. Paz , S. Wickenburg , H. Z. Tsai , D. G. De Oteyza , A. J. Bradley , M. M. Ugeda , P. Gorman , H. S. Jung , M. F. Crommie , A. Rubio , F. R. Fischer , Nat. Chem. 2016, 8, 678–683. 27325094 10.1038/nchem.2506

[13] B. Cirera , N. Giménez-Agulló , J. Björk , F. Martínez-Peña , A. Martin-Jimenez , J. Rodriguez-Fernandez , A. M. Pizarro , R. Otero , J. M. Gallego , P. Ballester , J. R. Galan-Mascaros , D. Ecija , Nat. Commun. 2016, 7, 11002. 26964764 10.1038/ncomms11002 PMC4793044

[14] G. Otero , G. Biddau , C. Sánchez-Sánchez , R. Caillard , M. F. López , C. Rogero , F. J. Palomares , N. Cabello , M. A. Basanta , J. Ortega , J. Méndez , A. M. Echavarren , R. Pérez , B. Gómez-Lor , J. A. Martín-Gago , Nature 2008, 454, 865–868. 18704082 10.1038/nature07193

[15] K. Amsharov , N. Abdurakhmanova , S. Stepanow , S. Rauschenbach , M. Jansen , K. Kern , Angew. Chem. Int. Ed. 2010, 49, 9392–9396; 10.1002/anie.201005000 21031392

[16] C. Sánchez-Sánchez , J. I. Martínez , V. Lanzilotto , G. Biddau , B. Gómez-Lor , R. Pérez , L. Floreano , M. F. López , J. Á. Martín-Gago , Nanoscale 2013, 5, 11058–11065. 24071968 10.1039/c3nr03706a

[17] L. Talirz , P. Ruffieux , R. Fasel , Adv. Mater. 2016, 28, 6222–6231. 26867990 10.1002/adma.201505738

[18] M. Batzill , Surf. Sci. Rep. 2012, 67, 83–115.

[19] K. A. Simonov , N. A. Vinogradov , A. S. Vinogradov , A. V. Generalov , E. M. Zagrebina , G. I. Svirskiy , A. A. Cafolla , T. Carpy , J. P. Cunniffe , T. Taketsugu , A. Lyalin , N. Mårtensson , A. B. Preobrajenski , ACS Nano 2015, 9, 8997–9011. 26301684 10.1021/acsnano.5b03280

[20] C. Sánchez-Sánchez , T. Dienel , O. Deniz , P. Ruffieux , R. Berger , X. Feng , K. Müllen , R. Fasel , ACS Nano 2016, 10, 8006–8011. 27428831 10.1021/acsnano.6b04025

[21] C. Sánchez-Sánchez , J. I. Martínez , N. Ruiz Del Arbol , P. Ruffieux , R. Fasel , M. F. López , P. L. De Andres , J. Á. Martín-Gago , J. Am. Chem. Soc. 2019, 141, 3550–3557. 30623650 10.1021/jacs.8b12239 PMC6459369

[22] M. Treier , C. A. Pignedoli , T. Laino , R. Rieger , K. Müllen , D. Passerone , R. Fasel , Nat. Chem. 2011, 3, 61–67. 21160519 10.1038/nchem.891

[23] M. Corso , W. Auwärter , M. Muntwiler , A. Tamai , T. Greber , J. Ostwalder , Science 2004, 303, 217–221. 14716010 10.1126/science.1091979

[24] J. Cai , P. Ruffieux , R. Jaafar , M. Bieri , T. Braun , S. Blankenburg , M. Muoth , A. P. Seitsonen , M. Saleh , X. Feng , K. Müllen , R. Fasel , Nature 2010, 466, 470–473. 20651687 10.1038/nature09211

[25] L. Liu , A. Corma , Chem. Rev. 2018, 118, 4981–5079. 29658707 10.1021/acs.chemrev.7b00776 PMC6061779

[26] Q. Sun , R. Zhang , J. Qiu , R. Liu , W. Xu , Adv. Mater. 2018, 30, 1705630. 10.1002/adma.201705630 29513368

[27] J. Neugebohren , D. Borodin , H. W. Hahn , J. Altschäffel , A. Kandratsenka , D. J. Auerbach , C. T. Campbell , D. Schwarzer , D. J. Harding , A. M. Wodtke , T. N. Kitsopoulos , Nature 2018, 558, 280–283. 29899477 10.1038/s41586-018-0188-x

[28] M. Giesen , Prog. Surf. Sci. 2001, 68, 1–153.

[29] N. Lin , D. Payer , A. Dmitriev , T. Strunskus , C. Wöll , J. V. Barth , K. Kern , Angew. Chem. Int. Ed. 2005, 44, 1488–1491; 10.1002/anie.200461390 15678430

[30] L. Dong , P. N. Liu , N. Lin , Acc. Chem. Res. 2015, 48, 2765–2774. 26317241 10.1021/acs.accounts.5b00160

[31] J. Eichhorn , T. Strunskus , A. Rastgoo-Lahrood , D. Samanta , M. Schmittel , M. Lackinger , Chem. Commun. 2014, 50, 7680–7682. 10.1039/c4cc02757d 24899567

[32] Q. Fan , T. Wang , L. Liu , J. Zhao , J. Zhu , J. M. Gottfried , J. Chem. Phys. 2015, 142, 101906. 25770495 10.1063/1.4906214

[33] Q. Li , B. Yang , J. Björk , Q. Zhong , H. Ju , J. Zhang , N. Cao , Z. Shi , H. Zhang , D. Ebeling , A. Schirmeisen , J. Zhu , L. Chi , J. Am. Chem. Soc. 2018, 140, 6076–6082. 29543446 10.1021/jacs.7b12278

[34] K. Niu , H. Lin , J. Zhang , H. Zhang , Y. Li , Q. Li , L. Chi , Phys. Chem. Chem. Phys. 2018, 20, 15901–15906. 29850686 10.1039/c8cp02013b

[35] C. Bronner , J. Björk , P. Tegeder , J. Phys. Chem. C 2015, 119, 486–493.

[36] J. Björk , J. Phys. Chem. C 2016, 120, 21716–21721.

[37] J. Mielke , F. Hanke , M. V. Peters , S. Hecht , M. Persson , L. Grill , J. Am. Chem. Soc. 2015, 137, 1844–1849. 25494667 10.1021/ja510528x

