# Supplementary material for: The Role of Metal Adatoms in a Surface‐Assisted Cyclodehydrogenation Reaction on a Gold Surface
Source: Angew Chem Int Ed Engl. 2022 Nov 8;61(49):e202212354. doi: 10.1002/anie.202212354 (PMC10099662; doi:10.1002/anie.202212354)
Supplement: Supplementary file 1 — Supporting Information [file ANIE-61-0-s001.pdf]

Angewandte Chemie
Eine Zeitschrift der Gesellschaft Deutscher Chemiker

Supporting Information

The Role of Metal Adatoms in a Surface-Assisted Cyclodehydrogenation Reaction on a Gold Surface

J. Björk, C. Sánchez-Sánchez*, Q. Chen, C. A. Pignedoli, J. Rosen, P. Ruffieux, X. Feng, A. Narita*, K. Müllen, R. Fasel*

S1. STM and nc-AFM experimental details

Experiments have been carried out in an UHV system ($P_{base} < 1 \times 10^{-10}$ mbar) equipped with a combined low-temperature STM and AFM (LT-STM/Qplus, Scienta Omicron). A Au(111) single crystal (SPL) was cleaned by iterative sputtering (Ar^+, 1 kV) and annealing (750 K) cycles until an atomically clean surface was observed by STM. 2,9-dibromo-7,14-diphenylbenzo[k]tetraphene precursor monomers were thermally sublimed from a 6-fold evaporator onto the clean Au(111) at a typical deposition rate of 1Å/min achieved at a molecular temperature of 450 K.

STM images were recorded either at LN_2 or LHe temperature (as stated in each figure together with the scanning parameters) in the constant current mode using electrochemically etched W tips. Non-contact AFM measurements were carried out using an electrochemically-etched W tip attached to a Qplus tuning fork sensor. To achieve the intramolecular resolution, the W tip was in-situ functionalized with a CO molecule "picked up" from the previously dosed surface.[1] Then, the sensor was excited at its resonance frequency (27160Hz) using a typical constant amplitude in the range of 70-80 pm. Then, the frequency shift was recorded, in the constant height mode, utilizing both the Omicron Matrix electronics and a HF2Li PLL by Zurich Instruments. All STM and AFM data reported in this study were processed with WaveMetrics Igor Pro software.

S2. Thermal evolution of polymer samples

The thermal annealing of polymer **2** was performed by elevating the sample temperature in three subsequent steps. (i) annealing at 525 K for 15 minutes; (ii) annealing at 575 K for 15 minutes; and (iii) annealing at 625 K for 15 minutes. After each annealing step, the surface was imaged by STM. Polymers **2** tend to agglomerate into islands due to attractive interactions between interdigitated phenyl rings. As temperature is increased, CDH is triggered and some of the phenyl rings planarize, inhibiting interpolymeric interdigitation and breaking up the islands. This behavior is evidenced in Figure S1.

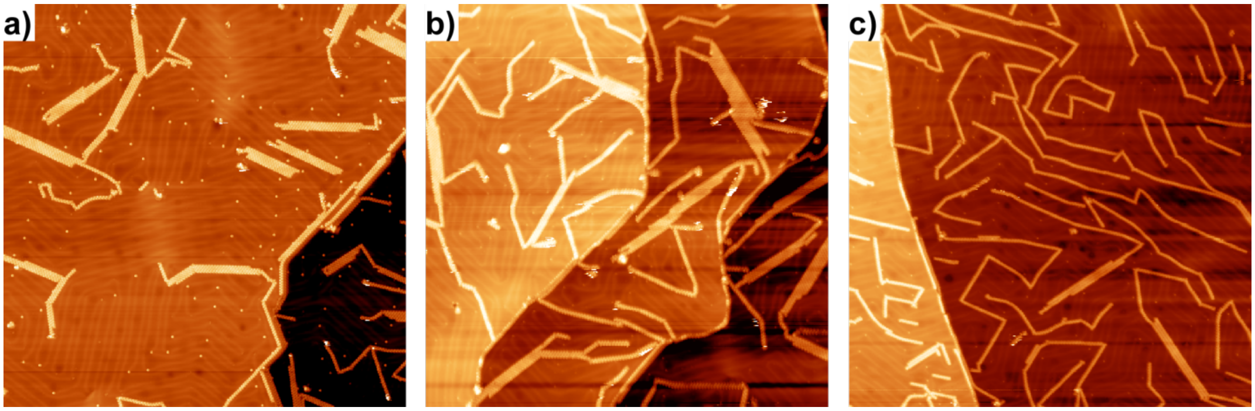

Figure S1 Evolution of the polymer arrangement with temperature. (**a)** At 475 K, most of the polymers are forming islands. STM parameters: (150 nm x 150 nm) I = 30 pA, V = -1.5 V. (**b)** At 525 K, polymers start to detach from the islands as a consequence of planarization of the external phenyl rings. STM parameters: (150 nm x 150 nm) I = 30 pA, V = -1.0 V. **c)** At 575 K, almost all polymers are isolated on the surface. STM parameters: (150 nm x 150 nm) I = 30 pA, V = 1.2 V.

While at 475 K (Figure S1a) most of the polymers are in islands, already at 525 K (Figure S1b) we observe a clear increase in the number of individual polymers. This effect is even more pronounced if the temperature is increased to 575 K (Figure S1c), where the vast majority of the polymers are isolated. This polymer separation as a result of molecular planarization is demonstrated in Figure S2. As we can see, for example, in the region highlighted in blue, some of the molecules in the polymers do no longer present the characteristic bright features at the edges associated to the out-of-plane tilting of the external phenyl rings. This is a clear indication of molecular cyclodehydrogenation.

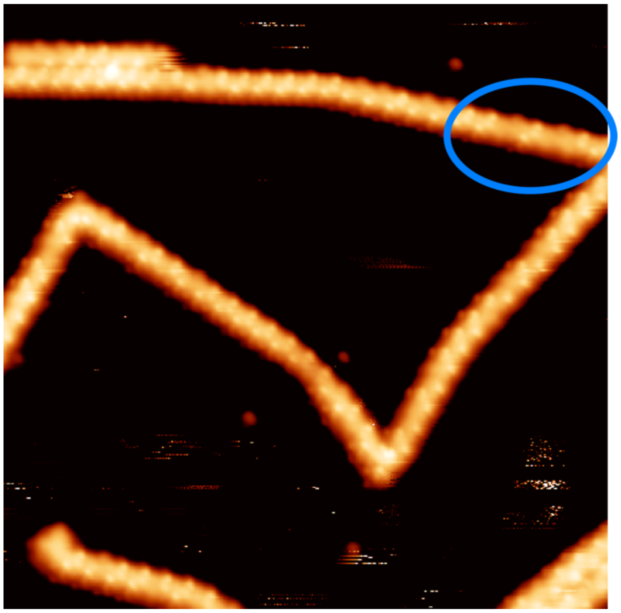

Figure S2. STM image of the polymers after annealing at 575 K reveal the coexistence of pristine (not cyclodehydrogenated) and of partially cyclodehydrogenated monomeric units within the same polymer strands. The blue ellipse highlights a cyclodehydrogenated region. STM parameters: (30 nm × 30 nm) I = 30 pA, V = 1.2 V, T = 78 K

S3. Structural model from STM images

As discussed in the main text, already from high-resolution STM images it is possible to observe discrepancies between the expected ribbon geometry (**3**) and the one of the actually obtained polymer **4**. Figure S3a shows one of such high-resolution STM images where the already mentioned kinked appearance becomes evident. If the two possible appropriately scaled schematic models of **3** and **4** are superimposed, one can clearly see that only the one comprising the formation of five-membered rings (**4**) fits (Figure S2b), while the one including six-membered rings (**3**) cannot reproduce the observed bays (Figure S2c). Thus, already from STM, we can conclude that the correct model is **4**.

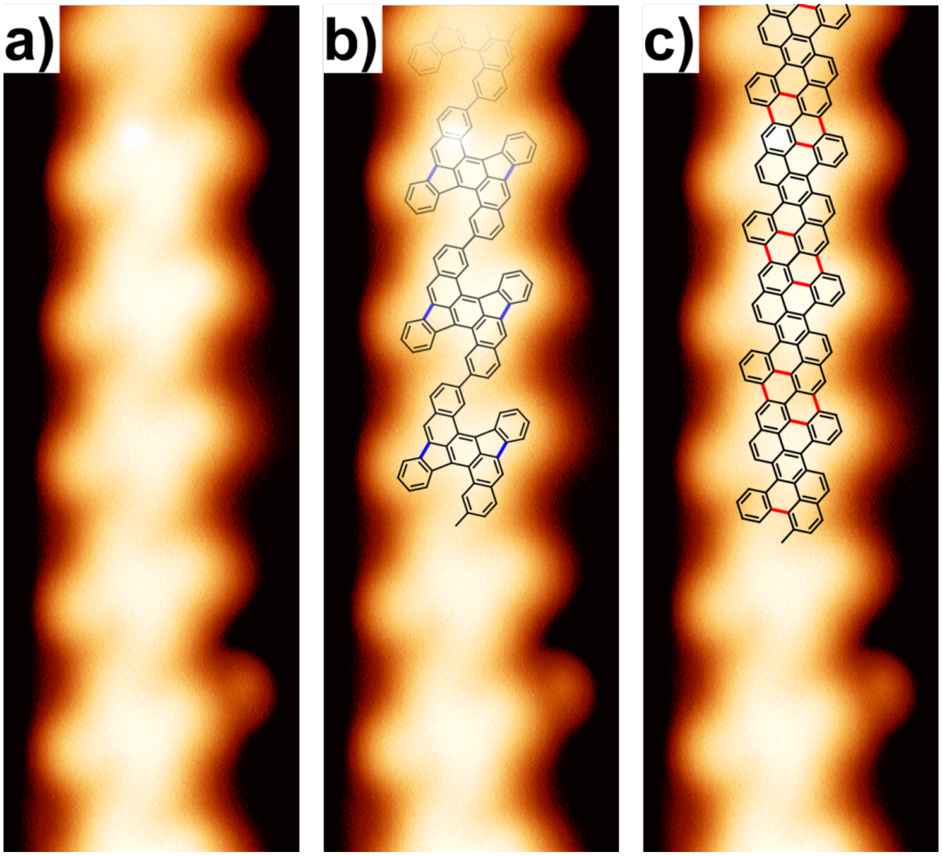

Figure S3 Extracting the correct structural model from high-resolution STM. (**a)** High-resolution STM image of the reaction product, revealing a kinked appearance. STM parameters: (2.5 nm x 7.0 nm) I = 2 pA, V = -0.1 V. (**b)** Same image than in panel (**a)** with model **4** superimposed (colored bonds correspond to newly formed ones). A perfect agreement is observed. **(c)** Same image than in panel (**a)** with model **3** superimposed (colored bonds correspond to newly formed ones). This model clearly does not fit with the STM image.

S4. Comparison of nc-AFM constant-height frequency-shift and tunneling current images acquired at different tip-sample heights with theoretical simulations.

Figure S4 shows a set of frequency-shift (top panels) and current (bottom panels) nc-AFM images obtained at different tip-sample heights, together with the corresponding theoretical simulations

considering both possible products **4** and **3** (5- and 6-membered rings, blue and red borders, respectively). The images obtained for the largest tip-sample separation are those shown in Figure S4a and b. At this height, we see some alternating bright maxima in the shape of horizontal lines in the frequency-shift image. These bright features indicate the presence of lifted regions within the edge of the structure under investigation as a result of cyclization of the external phenyl ring. According to the orientation of these bright lines, we can infer that the best match with simulations is for structure **4** involving the formation of 5-membered rings (note that although in the case of the 6-membered rings bright lines are also obtained, their orientation is completely different as they are rotated ~55° off the horizontal). When the tip-sample separation is reduced by 100 pm to try to revolve the internal structure of the polymer backbone, one can indeed differentiate some features of the internal structure in the frequency-shift images, like some of the hexagons in the backbone of the polymer (Figure S4c left). However, due to the strong interaction between the tip and the tilted terminal ring as a result of their close vicinity, we cannot achieve the desired internal resolution. On the other hand, the associated current image (Figure S4d left), which is not as much affected by the out-of-plane geometry of the structure as the frequency-shift one, already shows the presence of the six-membered rings and one can even discern the formation of extra rings at the expected position of the five-membered ones. In fact, if we compare both experimental images with the simulations, we observe a good agreement with structure **4**, while the agreement with the simulation for structure **3** is rather poor. Finally, in order to try to further improve the resolution of the polymer backbone, we have approached the tip by another 50 pm (panels e and f), although risking the integrity of the CO tip. At this separation, we get an improved resolution of the backbone but we cannot properly see either the five-membered rings or the adjacent tilted terminal benzene rings due to the strong repulsion which distorts the frequency-shift image (panel e left). However, one can better see the internal structure of the polymer in the current image (panel f left), confirming the formation of a new ring where the five-membered rings are expected. Once again, when the experimental data are compared with the corresponding simulations, one clearly observes that a good agreement is only achieved in the case of the non-benzenoid polymer **4**. Thus, thanks to these data, we can unambiguously state that the resulting structures correspond to the formation of polymers incorporating new five-membered rings as a consequence of cyclodehydrogenation of external phenyl rings.

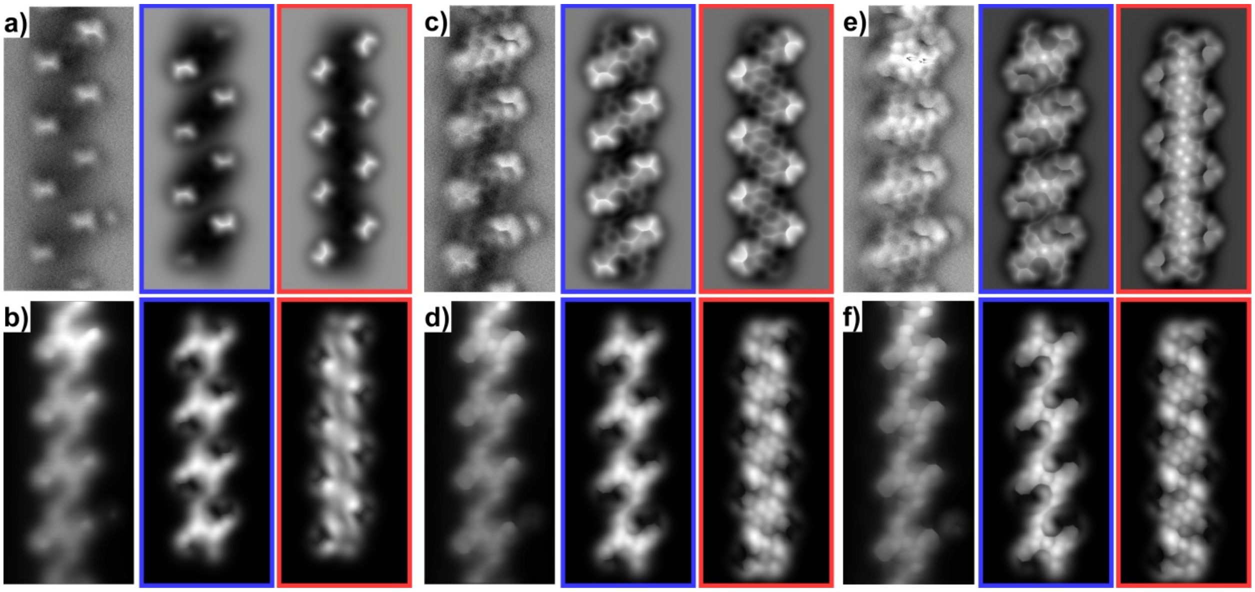

Figure S4 Set of frequency-shift (top panels) and current (bottom panels) images obtained with a CO-functionalized tip at different tip-sample separations. In each panel, the corresponding theoretical simulations associated to the five- (middle panel with blue border) and six-membered (right panel with red border) are also shown for comparison. Parameters: I = 2.0 pA, V = -0.1 V. 3 nm × 5.2 nm. **(a, b)** Images with the largest tip-sample separation. We have taken these images as "z" reference for the other images. $\Delta z = 0$. **(c, d)** Images obtained with $\Delta z = -100$ pm. **(e, f)** Images obtained with $\Delta z = -150$ pm.

S5. Statistical analysis of oligomer length

Figure S5 shows a length histogram for polymer **4** synthesized from precursor **1**. It reveals a broad length distribution from 10 to 40 nm, a small maximum at 50 nm, and a non-negligible number of oligomers with lengths exceeding 50 nm.

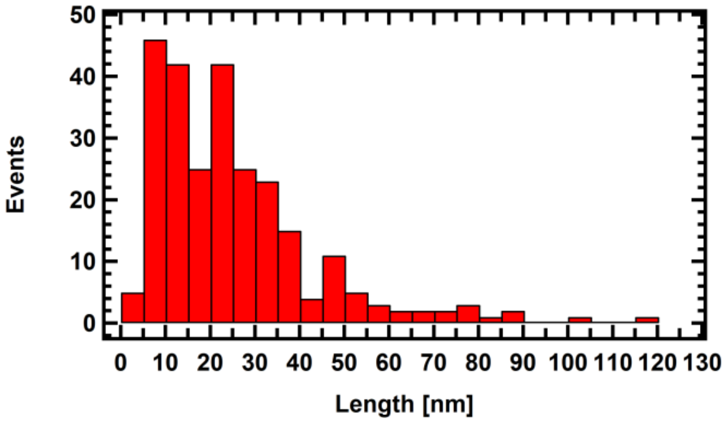

Figure S5 Polymer length histogram obtained from several large-scale images (150 nm x 150 nm) and a total number of events of 260.

To demonstrate the selectivity in the formation of polymer **4** against GNR **3**, we have also conducted a statistical analysis on the molecular products composing the final nanostructures. Figure S6 shows one of the STM images used in this analysis. As we can see, all the molecular units within the polymers present the same appearance and shape, which we have unambiguously assigned above to the formation of five-membered rings. In fact, our statistical analysis indicates that 93 % of the molecules have cyclodehydrogenated towards the five-membered ring, while the remaining 7 % corresponds to molecules at kinks and junctions, whose structure cannot be considered as they are the result of a wrong coupling at molecular positions different from the expected ones. Importantly, no molecular units exhibiting the formation of the six-membered rings are observed, in agreement with our model.

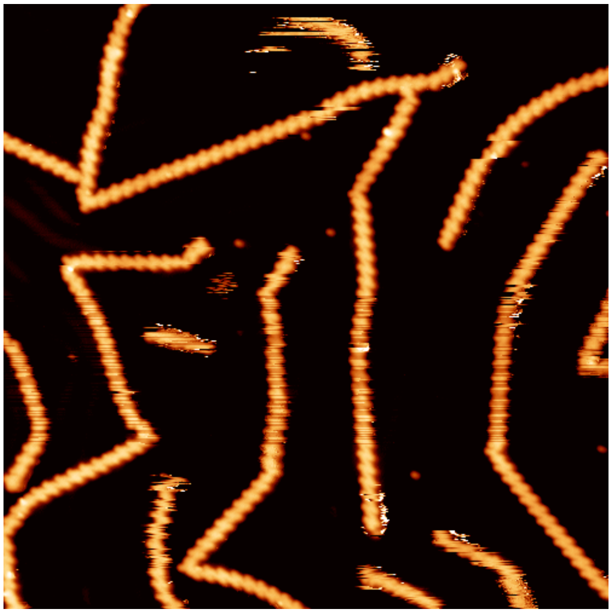

Figure S6 STM image confirming the selective formation of polymer **4** (composed of monomers having formed five-membered rings). STM parameters: (50 nm × 50 nm) I = 10 pA, V = 1.0 V, T = 78 K.

S6. Computational details

Reaction pathways were calculated with periodic density functional theory (DFT)-based transition state theory. The periodic DFT calculations were done with the VASP code,[2] employing projector-augmented wave potentials[3,4] and a plane wave basis set expanded to a kinetic energy cutoff of 400 eV. Exchange-correlation effects were described by the van der Waals density functional (vdW-DF),[5] using the version introduced by Hamada denoted rev-vdWDF2, which has been shown to describe the physisorption on Au(111) accurately.[6] The Au surfaces were modeled by a four layered slab separated by 15 Å of vacuum region. For the CDH reactions, we used a $p(8 \times 13)$ surface unit cell together with a 3×2 k-point sampling for polymer **2**, a $p(7 \times 7)$ surface unit cell together with a 3×3 k-point sampling for ortho-terphenyl. Transition states were calculated using a combination of the climbing-image nudged elastic band (CI-NEB)[7] and the Dimer method.[8] Local minima and transition states were optimized until the residual forces on all atoms, except the bottom two in the Au(111) slab (which were kept fixed), were smaller than 0.01 eV/Å.

Free energies for a particular state at temperature T and pressure p were calculated by

$$\Delta G(T,p) = \Delta H^{\text{elec}} - n_{ad}\mu_{\text{ad}} - \frac{1}{2} n_{\text{H}} T S_{\text{H}_2}(T,p), \tag{S1}$$

where ΔH^{elec} is the electronic enthalpy with respect to the initial state of the reaction (without adatom), n_{ad} the number of Au adatoms of the state under consideration and μ_{ad} the chemical potential of an Au adatom, n_{H} number of removed hydrogen atoms and $S_{\text{H}_2}(T,p)$ the entropy of the hydrogen gas at the given temperature and pressure, calculated as

$$S_{\text{H}_2}(T,p) = S_{\text{H}_2}(T,p_0) - k_{\text{B}} \ln\left(\frac{p}{p_0}\right), \tag{S2}$$

where $S_{\text{H}_2}(T,p_0)$ is the entropy of H_2 at standard pressure p_0, for which we used tabulated values,[9] and k_{B} is Boltzmann's constant. I.e., the free energy includes the energy of dissociatively desorbed H_2 molecules formed as a consequence of dehydrogenation. Regarding the chemical potential of the adatom, in the calculations with a single adatom we define it as a single adatom on the surface, *i.e.*, as

$$\mu_{\text{ad}} = H^{\text{elec}}_{\text{Au@Au(111)}} - H^{\text{elec}}_{\text{Au(111)}}, \tag{S3}$$

where $H^{\text{elec}}_{\text{Au@Au(111)}}$ is the electronic enthalpy of an isolated adatom on Au(111) and $H^{\text{elec}}_{\text{Au(111)}}$ is the electronic enthalpy of the Au(111) surface. In Figure 4 of the manuscript we show how the activation energy of dehydrogenation is affected by the entropy difference between a free adatom on the surface and an adatom interacting with a molecule (ΔS_{ad}). In this situation, the entropy contribution from this entropy difference is added to the chemical potential. That is, $-\Delta S_{\text{ad}} T$ is added to the chemical potential in Eq.

(S3), which has the result that for a state involving one adatom the free energy defined by Eq. (S1) is increased by $\Delta S_{\mathrm{ad}}T$.

For a particular state, the electron density difference was defined as the difference in electron density between the full system and the combined electron density between two subsystems with their coordinates frozen in the geometry of the full system: the Au adatom and Au(111) surface representing one subsystem and the molecular system the other. In this way, the electron density difference illustrates the rearrangement of charge due to the interaction of the molecular system with the adatom and the surface.

S7. Reaction pathway calculations and complementary calculated data

Here, we provide all the calculated pathways as well as estimates of reaction rates and dependency of dehydrogenation barriers on adatom entropy, which are the basis for the analysis presented in the manuscript.

CDH of polymer 2. For the calculations of the cyclodehydrogenation of polymer **2**, we considered an oligomer of three monomers, with the CDH taking place in the central part of such a trimer.

In Figure S7, two different ways of initiating the CDH by C-C coupling on the flat surface are compared. It is possible to form a five-membered ring by initial C-C coupling followed by dehydrogenation and tautomerization reactions, with an initial barrier of 1.85 eV. We were not able to isolate a pathway that leads to the formation of one six-membered ring by initial C-C coupling, but found a concerted pathway in which two six-membered rings are formed simultaneously. However, the barrier for initiating such a concerted process is relatively high (2.11 eV), and the steps for finalizing this reaction path were not considered further.

In Figures S8-S11, we provide the complete pathways of the reactions compared in Figure 3 in the manuscript, for which the reaction is initiated by dehydrogenation. For all pathways, both the electronic enthalpy at 0 K and the free energy obtained at a temperature of 600 K and a pressure of 10^{-12} bar, are provided. In Figure S8 and S9, the pathways for formation of a five- and six-membered ring, respectively, on the atomically flat surface are reported. In Figure S10 and S11 we show the corresponding pathways with the initial dehydrogenation step assisted by an Au adatom.

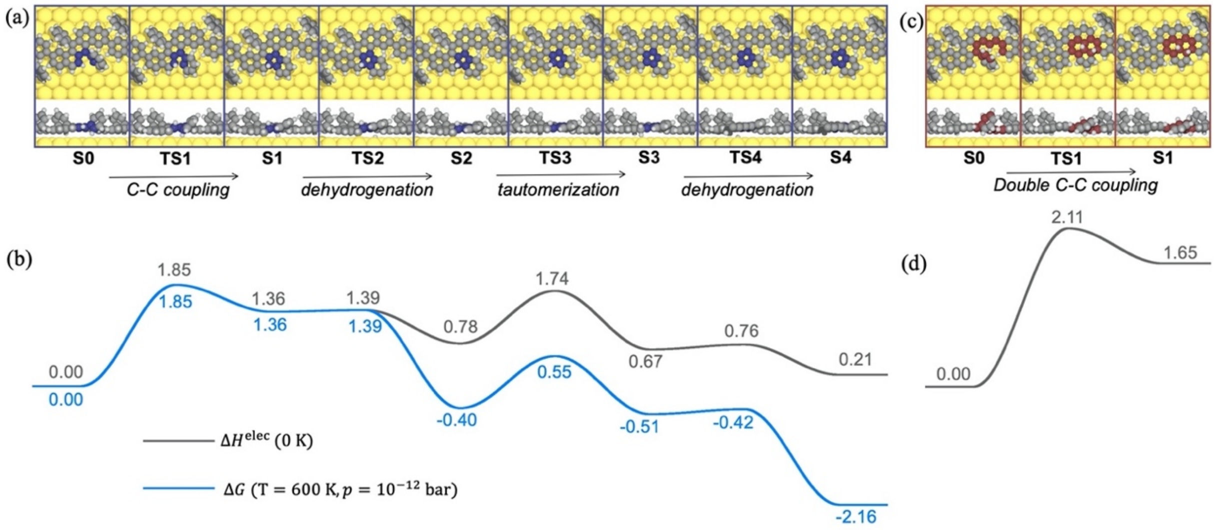

Figure S7 DFT-TST calculations of cyclodehydrogenation reactions initiated by C-C coupling on the flat Au(111) surface. (**a**,**b**) Reaction mechanism for the formation of a five-membered ring with (**a**) top and side views of both local minima (**S0** – **S4**) and transition states (**TS1** – **TS4**) and (**b**) comparison of the corresponding 0 K electronic enthalpy and free energy. (**c**,**d**) Initial step for the simultaneous formation of two six-membered rings. Units of energy profiles are in eV.

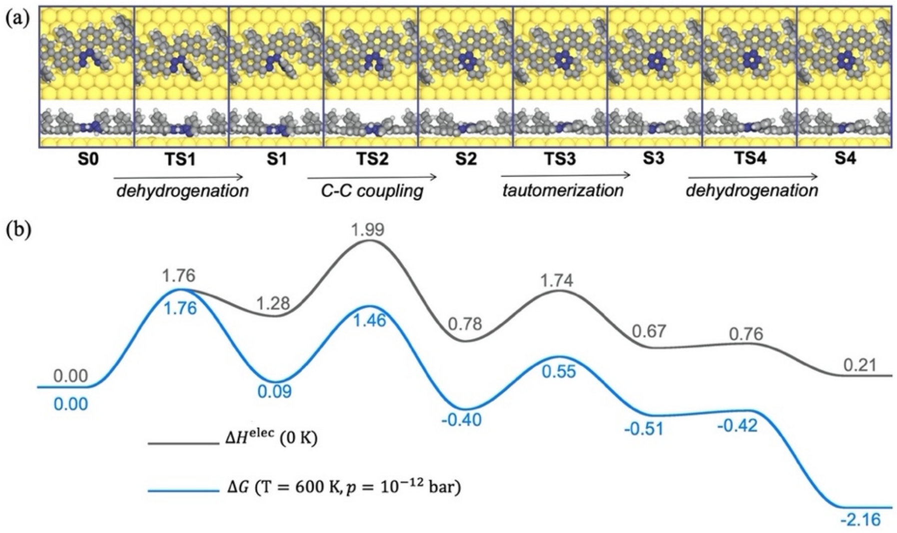

Figure S8 Formation mechanism for a CDH reaction resulting in a five-membered ring on the flat Au(111) surface initiated by dehydrogenation. (**a**) Top and side views of both local minima (**S0** – **S4**) and transition states (**TS1** – **TS4**) and (**b**) comparison of the corresponding 0 K electronic enthalpy and free energy. Units of energy profiles are in eV.

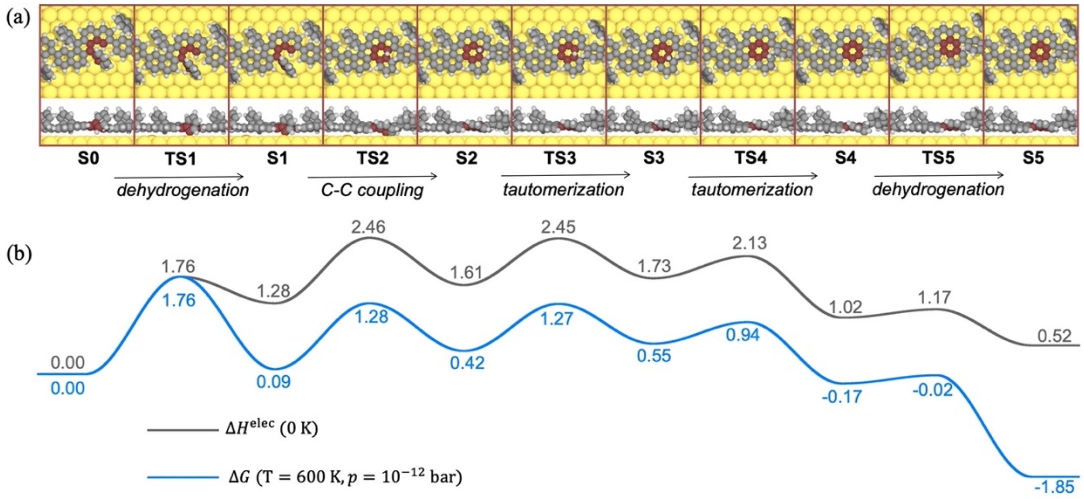

Figure S9 Formation mechanism for a CDH reaction resulting in a six-membered ring on the flat Au(111) surface initiated by dehydrogenation. (**a**) Top and side views of both local minima (**S0** – **S5**) and transition states (**TS1** – **TS5**) and (**b**) comparison of the corresponding 0 K electronic enthalpy and free energy. Units of energy profiles are in eV.

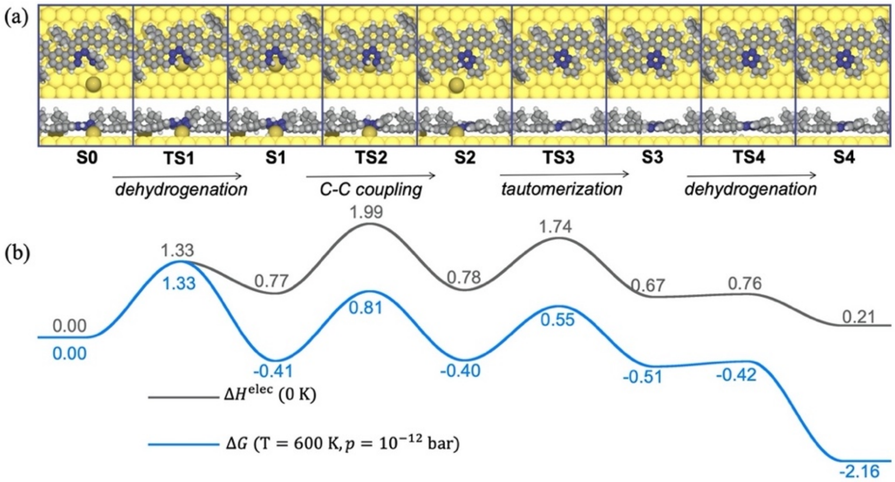

Figure S10 Formation mechanism for a CDH reaction resulting in a five-membered ring initiated by dehydrogenation activated by an Au adatom. (**a**) Top and side views of both local minima (**S0** – **S4**) and transition states (**TS1** – **TS4**) and (**b**) comparison of the corresponding 0 K electronic enthalpy and free energy. Units of energy profiles are in eV.

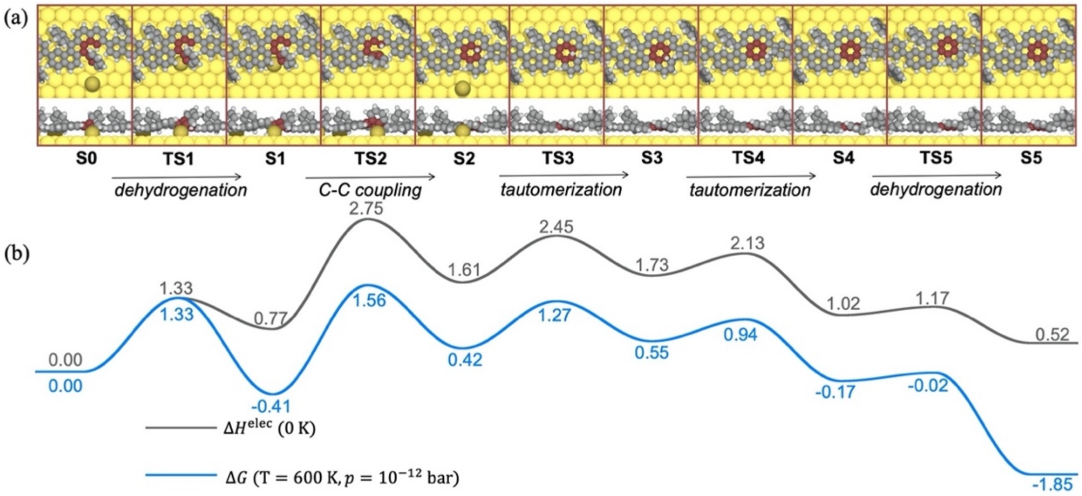

Figure S11 Formation mechanism for a CDH reaction resulting in a six-membered ring activated by an Au adatom.
(**a**) Top and side views of both local minima (**S0** – **S5**) and transition states (**TS1** – **TS5**) and (**b**) comparison of the corresponding 0 K electronic enthalpy and free energy. Units of energy profiles are in eV.

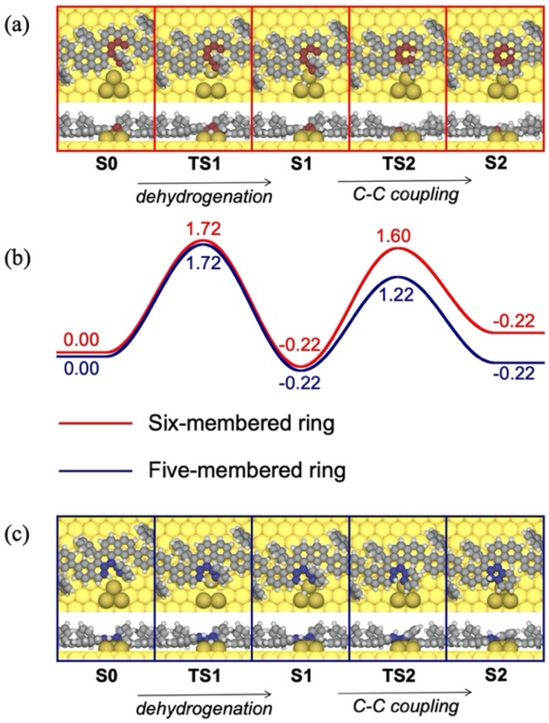

Figure S12 Formation mechanisms for the two initial steps of CDH reactions resulting in either a six-membered ring or five-membered ring, when activated by a cluster of three Au adatoms. (**a,c**) Top and side views of both local minima (**S0** – **S2**) and transition states (**TS1** – **TS2**) for (**a**) six-membered ring formation and (**c**) five-membered ring formation, and (**b**) the corresponding free energy profiles evaluated for $T = 600$ K and $p = 10^{-12}$ bar. The free energy profile was calculated with Eq. (S1) using chemical potential of an adatom defined as $\mu_{\mathrm{ad}} = \left(H^{\mathrm{elec}}_{\mathrm{3Au@Au(111)}} - H^{\mathrm{elec}}_{\mathrm{Au(111)}}\right)/3$, where $H^{\mathrm{elec}}_{\mathrm{3Au@Au(111)}}$ is the electronic enthalpy of an Au trimer on Au(111) and $H^{\mathrm{elec}}_{\mathrm{Au(111)}}$ is the electronic enthalpy of Au(111). Units of energy profiles are in eV.

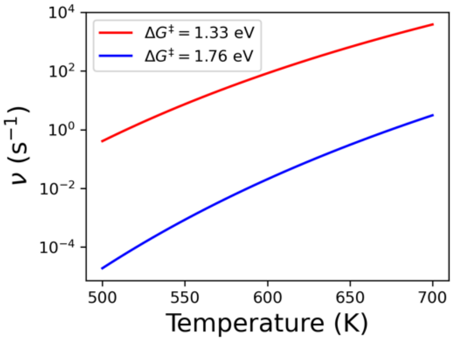

Figure S13 Reaction rates as a function of temperature calculated with the Eyring equations for activation energies corresponding to dehydrogenation with adatom (1.33 eV) and without adatom (1.76 eV).

Activation free energies as function of adatom entropy difference. It is of interest to understand under which conditions the adatom-assisted dehydrogenation can be anticipated to dominate over the dehydrogenation without adatom. For this, we also estimated the free energy activation energies by taking into account the entropy difference of an adatom moving freely on the surface and the adatom bonded to the molecule, which we denote as ΔS_{ad}, by adding the term $-\Delta S_{\mathrm{ad}}T$ to the reference energy of the adatom. I.e., the activation energy of the dehydrogenation with adatom is increased by the factor $\Delta S_{\mathrm{ad}}T$, while the activation energy without adatom does not depend on this factor. The results are summarized in Figure S14, demonstrating that the activation free energy is lower for the adatom-assisted dehydrogenation up to $\Delta S_{\mathrm{ad}} = 0.72$ meV/K. According to the Sackur-Tetrode equation in 2D,[10] such a value of the entropy is obtained for a 2D gas of Au atoms for a concentration of one atom per 4 $Å^2$, which is even higher than the concentration of surface atoms of Au(111). However, the Sackur-Tetrode equation gives an upper limit of the translational entropy of the adatoms diffusing over the surface, valid if they can be regarded as an ideal 2D gas. Furthermore, in ΔS_{ad} the entropy of a free adatom on the surface needs to be balanced with the entropy of an adatom interacting with the molecular system. To understand all details of the free energy of the system is, however, not conceivable within our computational framework. However, we would like to stress that our conclusion that CDH is driven by adatoms is based on the strong preference of five-membered ring formation, which is only explained by the participation of adatoms.

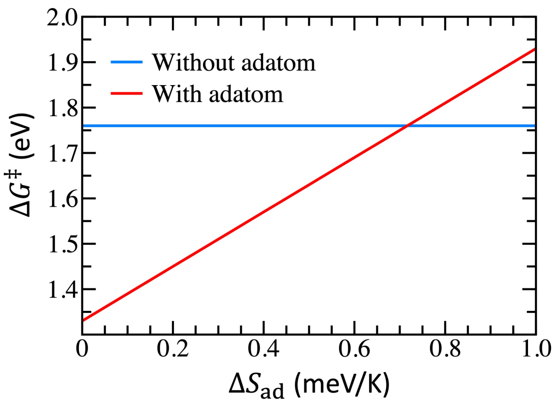

Figure S14 Activation energies of initial dehydrogenation ($\Delta G^{\ddagger}$) as a function of adatom entropy difference (ΔS_{ad}). The adatom entropy difference should be interpreted as the entropy difference of an Au adatom freely diffusing over the surface and an Au adatom bonded to the polymer, which adds a factor $-\Delta S_{\mathrm{ad}}T$ to the chemical potential of the adatom in Eq. (S1) of the Supporting Information. The activation free energy was calculated as $\Delta G^{\ddagger} = \Delta H^{\ddagger} + \Delta S_{\mathrm{ad}}T$ for the case with an adatom (the entropy term lowers the energy of the initial state), where $\Delta H^{\ddagger}$ is taken as the potential energy barrier obtained from the DFT-TST calculations. For the dehydrogenation without adatom the entropy of the adatom does not affect the activation energy.

CDH of *ortho*-terphenyl

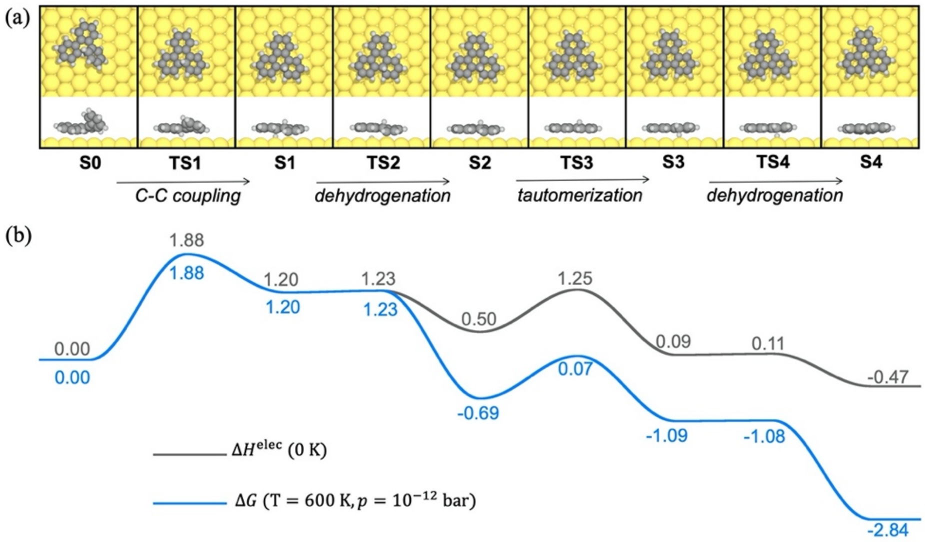

Figure S15 Mechanism of CDH of terphenyl into triphenylene initiated by C-C coupling on the flat Au(111) surface. (**a**) Top and side views of both local minima (**S0** – **S4**) and transition states (**TS1** – **TS4**) and (**b**) comparison of the corresponding 0 K electronic enthalpy and free energy. Units of energy profiles are in eV.

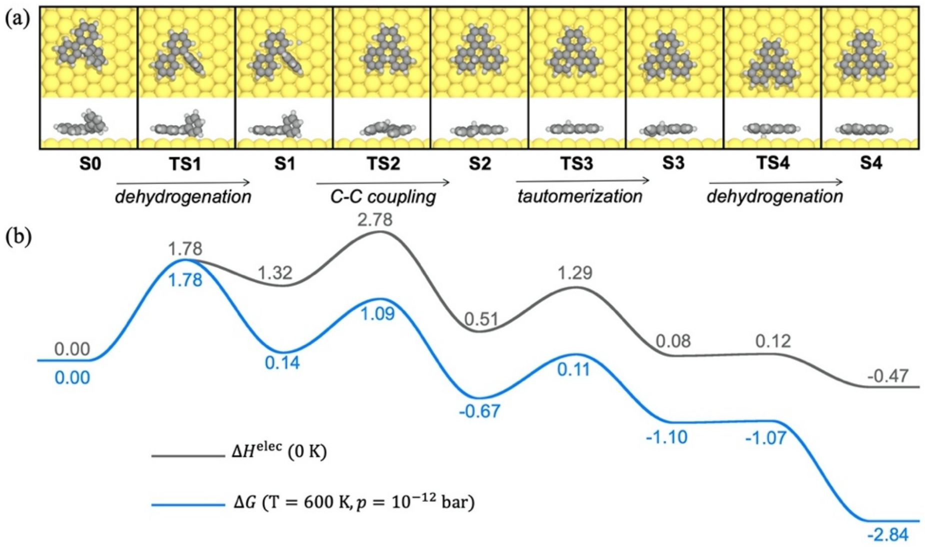

Figure S16 Mechanism of CDH of terphenyl into triphenylene initiated by dehydrogenation on the flat Au(111) surface. (**a**) Top and side views of both local minima (**S0** – **S4**) and transition states (**TS1** – **TS4**) and (**b**) comparison of the corresponding 0 K electronic enthalpy and free energy. Units of energy profiles are in eV.

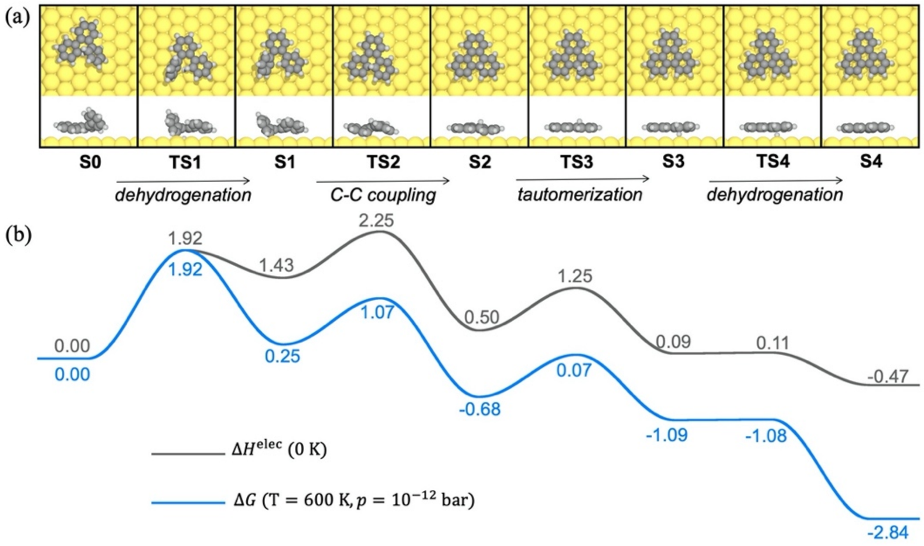

Figure S17 Alternative mechanism of CDH of terphenyl into triphenylene initiated by dehydrogenation on the flat Au(111) surface. (**a**) Top and side views of both local minima (**S0** – **S4**) and transition states (**TS1** – **TS4**) and (**b**) comparison of the corresponding 0 K electronic enthalpy and free energy. Units of energy profiles are in eV.

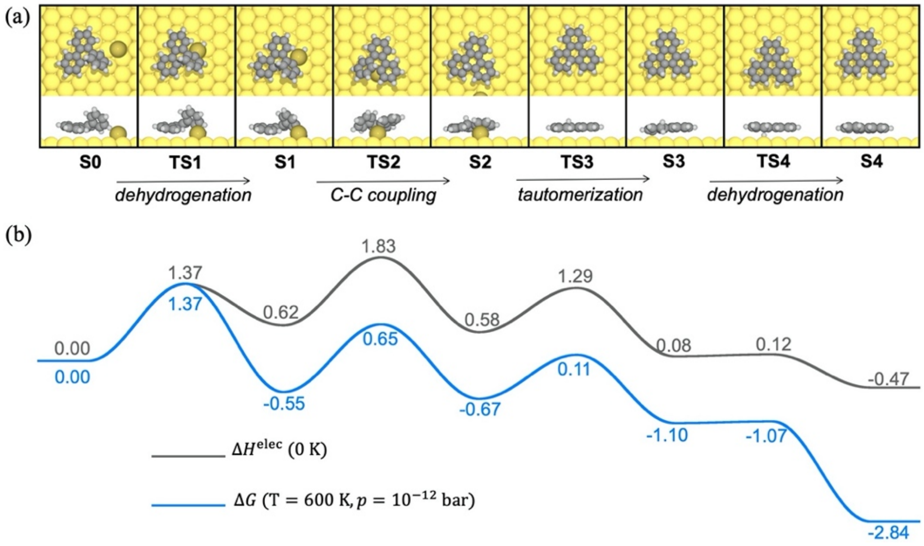

Figure S18 Mechanism of CDH of terphenyl into triphenylene initiated by dehydrogenation supported by an Au adatom. (**a**) Top and side views of both local minima (**S0** – **S4**) and transition states (**TS1** – **TS4**) and (**b**) comparison of the corresponding 0 K electronic enthalpy and free energy. Note that the C-C coupling (**S1**–**S2**) is a two-step process, as the phenyl group connected to the adatom first need to undergo a rotation. Only the efficient barrier, defined by the energy of the transition state for the actual coupling, is shown. Units of energy profiles are in eV.

S8. STM and nc-AFM simulations

We used DFT to obtain the nc-AFM and HRSTM simulations combining the CP2K code[11] and the probe particle code.[12] The simulations were done using the AiiDAlab platform.[13] The microscopy simulations were obtained for the equilibrium geometry of finite (four precursor units) segments of the polymers adsorbed on gold. We used simulation cells consisting of four atomic layers of Au along the [111] direction. A layer of hydrogen atoms was used to passivate one side of the slab to suppress the Au(111) surface state. 40 Å of vacuum was included in the simulation cell to decouple the system from its periodic replicas in the direction perpendicular to the surface. The electronic states were expanded with a TZV2P Gaussian basis set[14] for C and H species and a DZVP basis set for Au species. A cutoff of 600 Ry was used for the plane-wave basis set. Norm-conserving Goedecker-Teter-Hutter pseudopotentials[15] were used to represent the frozen core electrons of the atoms. We used the PBE parameterization for the generalized gradient approximation of the exchange-correlation functional.[16] To account for van der Waals interactions, we used the D3 scheme proposed by Grimme.[17] The gold surface was modeled using a supercell of size 29.48×66.38 Å^2 (corresponding to 1140 Au atoms). To obtain the equilibrium geometries, we kept the atomic positions of the bottom two layers of the slab fixed to the ideal bulk positions, and all other atoms were relaxed until forces were lower than 0.005 eV/Å.

S9. Monomer synthesis and characterization

General methods

All reactions working with air- or moisture- sensitive compounds were carried out under argon atmosphere using standard Schlenk line techniques. Unless otherwise noted, all starting materials were purchased from commercial sources and used without further purification. All other reagents were used as received. Thin layer chromatography (TLC) was done on silica gel coated aluminum sheets with F254 indicator and column chromatography separation was performed with silica gel (particle size 0.063 – 0.200 mm). Melting points were determined on a Büchi hot stage apparatus and were uncorrected. Nuclear Magnetic Resonance (NMR) spectra were recorded using Bruker DPX 300 or 250 MHz spectrometers. Chemical shifts (δ) were expressed in ppm relative to the residual signals of deuterated solvent (Methylene Chloride-d_2, ^{1}H: 5.32 ppm, ^{13}C: 53.84 ppm). Abbreviations: s = singlet, d = doublet, dd = double doublet, t = triplet, m = multiplet. Coupling constants (J) were recorded in Hertz. Field desorption mass (FD-MS) spectra were measured using a VG instruments ZAB 2-SE-FPD using 8 kV accelerating voltage. Accurate mass measurements were performed on a SYNAPT G2-Si high definition Q-TOF mass spectrometer (Waters Corp., Manchester, UK) by matrix-assisted laser desorption/ionization (MALDI). The instrument was operated at a resolution of 20 000 (M/ΔM) and calibrated by clusters of red phosphorous in a mass range of 100 to 8000 Da. Data processing was done with MassLynx software V4.1. Or on a G6545A Q-ToF (Agilent GmbH, Waldbronn, Germany) with atmospheric pressure chemical ionization (APCI) mass spectrometer. Single crystal diffraction data was collected on a STOE IPDS 2T diffractometer with Mo-K_α radiation.

Synthetic routes

The synthesis of monomer **1** is shown in the supplementary scheme S1 below. Bispinacolborate **4** was synthesized from 1,4-dibromo-2,5-diiodobenzene (**2**) by twofold selective Suzuki coupling with phenylboronic acid, followed by Miyaura borylation. 4-Bromo-1-iodo-2-nitrobenzene (**6**) was obtained by Sandmeyer iodination of 4-bromo-2-nitroaniline (**5**) in 64% yield, and the nitro group was reduced using iron powder to give 5-bromo-2-iodoaniline (**7**) in a quantitative yield. Subsequently, **7** was transformed to 5-bromo-2-{(triisopropylsilyl)ethynyl}aniline (**8**) through a Sonogashira coupling with triisopropylsilyl (TIPS)-acetylene in 87% yield. Afterwards, 4-bromo-2-iodo-1-(TIPS-ethynyl)benzene (**9**) was obtained by Sandmeyer iodination of **8** in 62% yield and further Suzuki coupling with bispinacolborate **4** afforded precursor **10** in a yield of 46%. Then, **10** was deprotected with tetra-n-butylammonium fluoride to give precursor **11** in 96% yield, which was cyclized with $PtCl_2$ in toluene at 120 °C to afford 2,9-dibromo-7,14-diphenylbenzo[k]tetraphene (**1**) in 21% yield.

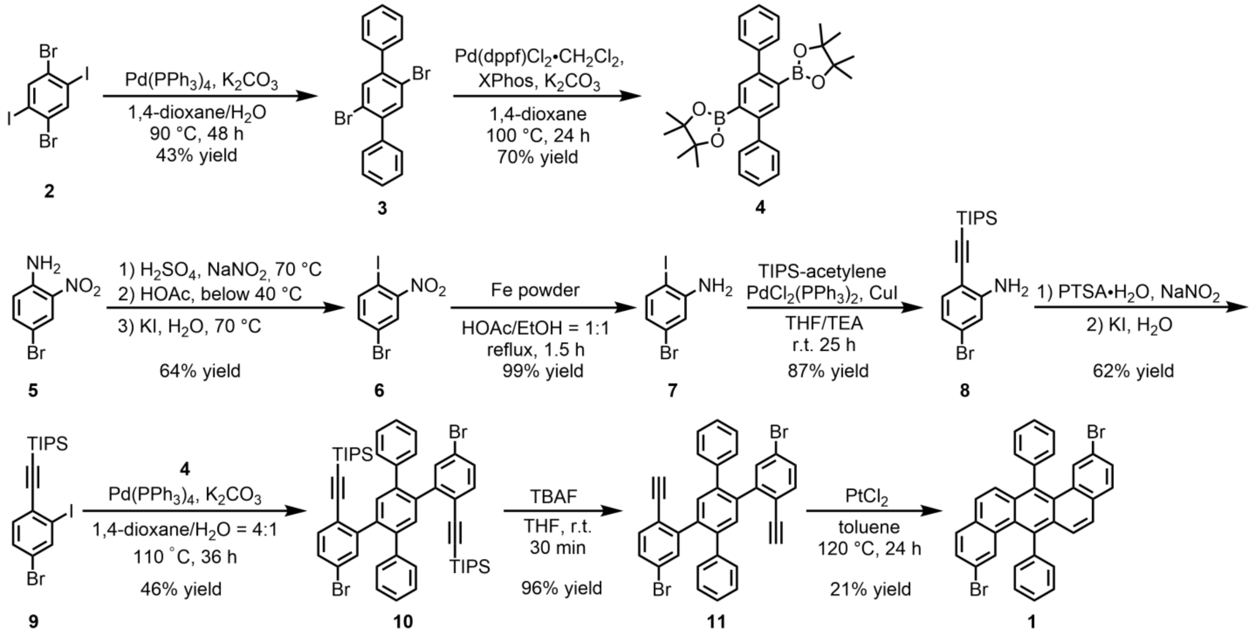

Scheme S1 Synthetic route towards 2,9-dibromo-7,14-diphenylbenzo[*k*]tetraphene (**1**)

Synthetic details

1,4-Dibromo-2,5-diphenylbenzene (**3**):[18]

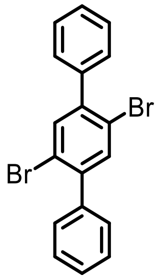

A 250-mL two-neck round-bottom flask equipped with a magnetic stirring bar was charged with 1,4-dibromo-2,5-diiodobenzene (**2**) (5.0 g, 10 mmol), phenylboronic acid (2.6 g, 21 mmol), K_2CO_3 (5.7 g, 41 mmol) and a mixture of 1,4-dioxane (100 mL) and H_2O (30 mL). The suspension was degassed by bubbling with argon for 20 min. Under nitrogen atmosphere, $Pd(PPh_3)_4$ (0.61 g, 0.52 mmol) was added and the mixture was degassed for another 10 min. After heating at 90 °C for 48 h, the reaction mixture was cooled down to room temperature and diluted with ethyl acetate (100 mL) and washed with water (50 mL). The aqueous phase was extracted twice with ethyl acetate (50 mL). The combined organic phases were washed with brine (50 mL), dried over Na_2SO_4 and evaporated. The obtained residue was recrystallized from cold ethyl acetate to afford the title compound (1.7 g, 43 % yield) as white solid. Mp: 210.7 – 211.8 °C; ^{1}H NMR (300 MHz, Methylene Chloride-*d*$_2$) δ 7.66 (s, 2H), 7.52 – 7.37 (m, 10H); ^{13}C NMR (75 MHz, Methylene Chloride-*d*$_2$) δ 143.4, 139.9, 135.6, 129.7, 128.6, 121.7; HRMS (APCI): Calcd for $C_{18}H_{12}Br_2$: 385.9306 $[M]^+$, found: 385.9304. The characterization data are consistent with the literature.[18]

1,4-Bis(4',4',5',5'-tetramethyl-1',3',2'-dioxaborolan-2-yl)-2,5-diphenylbenzene (**4**)

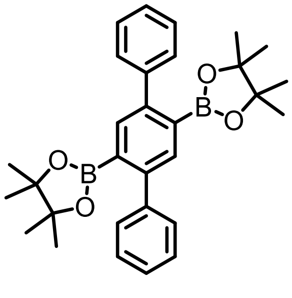

To a 250-mL two-neck round-bottom flask was added 1,4-dibromo-2,5-diphenylbenzene (**3**) (1.50 g, 3.86 mmol), bis(pinacolato)diboron (2.90 g, 11.6 mmol), $Pd(dppf)Cl_2{\cdot}CH_2Cl_2$ (158 mg, 0.193 mmol), 2-dicyclohexylphosphino-2′,4′,6′-triisopropylbiphenyl (184 mg, 0.386 mmol) and K_2CO_3 (3.20 g, 23.2 mmol). The flask was evacuated and backfilled with argon for three times before 1,4-dioxane (100 mL)

was added via a syringe, and then the mixture was degassed by bubbling with argon for 10 min. After heating at 100 °C for 24 h, the reaction mixture was cooled down to room temperature, diluted with ethyl acetate (100 mL) and washed with water (100 mL). After separation, the aqueous phase was extracted with ethyl acetate (50 mL) for three times. The combined organic layers were washed with brine (100 mL), dried over Na_2SO_4 and evaporated. The obtained residue was purified by column chromatography (eluent: *n*-hexane:ethyl acetate = 1:1) to afford the title compound (1.3 g, 70 % yield) as white solid. Mp: 296.6 – 297.9 °C; ^{1}H NMR (300 MHz, Methylene Chloride-*d*$_2$) δ 7.71 (s, 2H), 7.47 – 7.33 (m, 10H), 1.21 (s, 24H); ^{13}C NMR (75 MHz, Methylene Chloride-*d*$_2$) δ 145.5, 143.2, 135.5, 129.5, 128.3, 127.4, 84.2, 24.8; HRMS (APCI): Calcd for $C_{30}H_{36}B_2O_4$: 483.2872 $[M+H]^+$, found: 483.2883.

4-Bromo-1-iodo-2-nitrobenzene (**6**):[19]

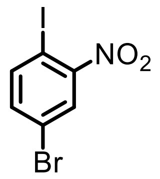

$NaNO_2$ (3.4 g, 49 mmol) was added gradually to concentrated H_2SO_4 (36 mL) at room temperature in a 500-mL round-bottom flask equipped with a stirring bar, and the resulting mixture was heated to 70 °C with stirring. After heating for 15 min, the mixture was cooled down to room temperature. 4-Bromo-2-nitroaniline (**5**) (9.6 g, 44 mmol) dissolved in glacial acetic acid (88 mL) was added dropwise to the above-mentioned solution while maintaining the temperature below 40 °C. After the addition, the resulting mixture was stirred at room temperature for another 30 min, and then poured into a solution of KI (8.0 g, 4.8 mmol) in water (80 mL) preheated to 70 °C. The mixture was stirred at this temperature for 20 min and then diluted with 600 mL of water. The precipitates were filtered, washed with water (200 mL) and dried under vacuum for 24 h to afford the title compound (12.1 g, 64 % yield) as light yellow solid. Mp: 88.6 – 88.9 °C; ^{1}H NMR (300 MHz, Methylene Chloride-*d*$_2$) δ 8.01 (d, J = 2.2 Hz, 1H), 7.92 (d, J = 8.4 Hz, 1H), 7.44 (dd, J = 8.3, 2.1 Hz, 1H); ^{13}C NMR (75 MHz, Methylene Chloride-*d*$_2$) δ 143.4, 137.0, 128.8, 123.0, 118.6, 84.7; FD-MS (8 kV): *m*/*z* 327.6. The characterization data are consistent with the literature.[19]

5-Bromo-2-iodoaniline (**7**):[20]

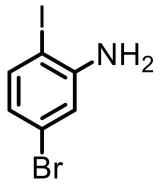

To a gently refluxing solution of 4-bromo-1-iodo-2-nitrobenzene (**6**) (15.0 g, 45.7 mmol) in glacial acetic acid (100 mL) and ethanol (100 mL) was added Fe powder (10 g, 18 mmol) in portions. After refluxing for another 1.5 h, the reaction mixture was cooled down to room temperature, and then filtered. Insoluble solids were washed with ethyl acetate (200 mL). The filtrate was washed with water (150 mL) and the aqueous phase was extracted twice with ethyl acetate (100 mL). The combined organic phases were washed with a saturated aqueous solution of Na_2CO_3 (200 mL) and brine (200 mL), dried over Na_2SO_4 and evaporated. The residue was purified by silica gel column chromatography (eluent: *n*-hexance:ethyl acetate = 10:1) to afford the title compound (13.5 g, 99% yield) as white solid. Mp: 53.8 – 54.5 °C; ^{1}H NMR (300 MHz, Methylene Chloride-d_2) δ 7.47 (d, J = 8.4 Hz, 1H), 6.89 (d, J = 2.2 Hz, 1H), 6.64 – 6.55 (dd, J = 8.4, 2.2 Hz, 1H), 4.21 (s, 2H); ^{13}C NMR (75 MHz, Methylene Chloride-d_2) δ 148.8, 140.4, 123.4, 122.9, 117.3, 82.0; HRMS (ESI-MS): *m*/*z* Calcd for C_6H_5BrIN: 297.8723 $[M+H]^+$, found: 297.8719. The characterization data are consistent with the literature.[20]

5-Bromo-2-{(triisopropylsilyl)ethynyl}aniline (**8**):

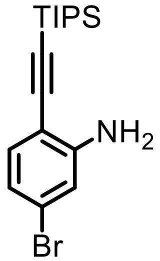

To a degassed solution of 5-bromo-2-iodoaniline (**7**) (3.0 g, 1.0 mmol), $Pd(PPh_3)_2Cl_2{\cdot}CH_2Cl_2$ (69 mg, 0.097 mmol) and CuI (39 mg, 0.017 mmol) in a mixture of tetrahydrofuran (60 mL) and triethylamine (9 mL) was added (triisopropylsilyl)acetylene (2.0 g, 11 mmol) at room temperature. After stirring at room temperature for 25 h, the reaction mixture was diluted with ethyl acetate (50 mL) and washed with water (50 mL). The aqueous phase was extracted with ethyl acetate (50 mL) for three times and the combined organic phases were washed with brine (50 mL), dried over Na_2SO_4 and evaporated. The residue was purified by silica gel column chromatography (eluent: *n*-hexane: ethyl acetate = 10:1) to give the title

compound (3.06 g, 87% yield) as colorless oil. ^{1}H NMR (300 MHz, Methylene Chloride-*d*$_2$) δ 7.15 (d, J = 8.2 Hz, 1H), 6.87 (d, J = 2.0 Hz, 1H), 6.77 (dd, J = 8.2, 2.0 Hz, 1H), 4.37 (s, 2H), 1.14 (s, 21H); ^{13}C NMR (75 MHz, Methylene Chloride-*d*$_2$) δ 150.1, 133.9, 124.0, 121.0, 117.2, 107.6, 103.2, 97.8, 19.0, 11.8; HRMS (APCI): Calcd for $C_{17}H_{26}BrNSi$: 351.1012 [M]$^+$, found: 351.1023.

4-Bromo-2-iodo-1-{(triisopropylsilyl)ethynyl}benzene (**9**):

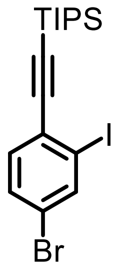

To a 250-mL two-neck round-bottom flask was added 5-bromo-2-{(triisopropylsilyl)ethynyl}aniline (**8**) (2.2 g, 6.2 mmol), *p*-TsOH·H_2O (3.56 g, 18.7 mmol) and acetonitrile (60 mL). The resulting suspension of amine salt was cooled down to 0 °C and a solution of $NaNO_2$ (0.86 g, 12 mmol) and KI (2.59 g, 15.6 mmol) in water (5 mL) was added dropwise to this mixture. The reaction mixture was stirred at 0 °C for 2 h, and then allowed to gradually warm up to room temperature. After stirring for another 2 h, a saturated aqueous solution of Na_2SO_3 (50 mL) was added, and then the mixture was diluted with ethyl acetate (100 mL) and washed with water. The aqueous phase was extracted twice with ethyl acetate (50 mL), and the combined organic layers were washed with brine (50 mL), dried over Na_2SO_4 and evaporated. The residue was purified by silica gel column chromatography (eluent: *n*-hexane) to give the title compound (1.8 g, 62% yield) as colorless oil. ^{1}H NMR (300 MHz, Methylene Chloride-*d*$_2$) δ 8.02 (d, J = 2.1 Hz, 1H), 7.45 (dd, J = 8.3, 2.1 Hz, 1H), 7.35 (d, J = 8.4 Hz, 1H), 1.16 (s, 21H); ^{13}C NMR (75 MHz, Methylene Chloride-*d*$_2$) δ 141.4, 134.4, 131.6, 129.7, 122.8, 107.6, 101.6, 97.5, 19.0, 11.9.

2',5'-Bis[5-bromo-2-{(triisopropylsilyl)ethynyl}phenyl]-1,1':4',1''-terphenyl (**10**):

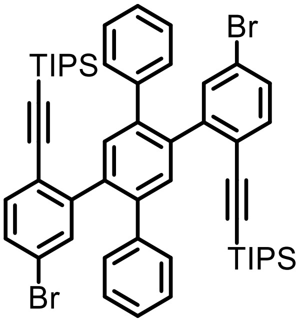

To a 50-mL Schlenk tube was added 1,4-bis(4',4',5',5'-tetramethyl-1',3',2'-dioxaborolan-2-yl)-2,5-diphenylbenzene (**4**) (400 mg, 0.829 mmol), 4-bromo-2-iodo-1-{(triisopropylsilyl)ethynyl}benzene (**9**) (845 mg, 1.82 mmol), $Pd(PPh_3)_4$ (95.9 mg, 83.0 μmol) and K_2CO_3 (0.687 g, 4.98 mmol). The flask was evacuated and backfilled with argon for three times. Then 1,4-dioxane/H_2O (16 mL/4 mL) was added using a syringe. The mixture was degassed by bubbling with argon for 10 min. After heating at 110 °C for 36 h, the reaction mixture was cooled down to room temperature, diluted with ethyl acetate (50 mL) and washed with water (50 mL). The aqueous phase was extracted twice with ethyl acetate (50 mL). The combined organic phases were washed with brine (50 mL), dried over Na_2SO_4 and evaporated. The obtained residue was purified by silica gel column chromatography (eluent: *n*-hexane:ethyl acetate =10:1) to afford the title compound (340 mg, 46% yield) as white solid. ^{1}H NMR (300 MHz, Methylene Chloride-d_2) δ 7.45 (s, 2H), 7.36 (s, 4H), 7.21 (d, J = 2.4 Hz, 12H), 0.95 (s, 42H); ^{13}C NMR (75 MHz, Methylene Chloride-d_2) δ 140.7, 140.1, 133.0, 130.5, 130.2, 128.3, 127.3, 122.1, 115.6, 105.6, 18.9, 11.8; HRMS (APCI): m/z Calcd for $C_{52}H_{60}Br_2Si_2$: 898.2600 [M]$^+$, found: 898.2582.

2',5'-Bis(5-bromo-2-ethynylphenyl)-1,1':4',1''-terphenyl (**11**):

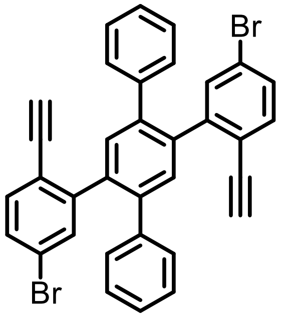

2',5'-Bis[5-bromo-2-{(triisopropylsilyl)ethynyl}phenyl]-1,1':4',1''-terphenyl (**10**) (400 mg, 0.444 mmol) was dissolved in tetrahydrofuran (12 mL) and the solution was degassed by argon bubbling for 10 min. To the solution was added tetra-*n*-butylammonium fluoride (0.44 mL, 1.0 M in tetrahydrofuran, 0.44 mmol)

dropwise using a syringe. After 30 min, the reaction was quenched by addition of methanol (100 mL). The precipitate was collected by filtration and washed with methanol (30 mL) to afford the title compound (250 mg, 96% yield) as white solid. ^{1}H NMR (300 MHz, Methylene Chloride-d_2) δ 7.52 (s, 2H), 7.37 (d, J = 5.5 Hz, 5H), 7.23 (s, 9H), 3.13 (s, 2H); ^{13}C NMR (75 MHz, Methylene Chloride-d_2) δ 146.4, 140.7, 140.3, 138.1, 134.9, 134.3, 133.4, 130.7, 130.3, 128.5, 127.4, 122.9, 121.5, 82.4, 82.0; HRMS (MALDI-TOF): m/z Calcd for $C_{34}H_{20}Br_2$: 585.9932 $[M]^+$, found: 585.9949.

2,9-Dibromo-7,14-diphenylbenzo[*k*]tetraphene (**1**):

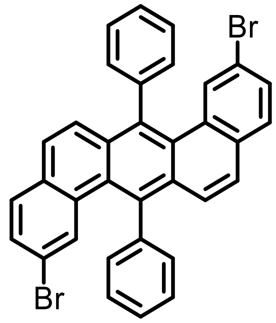

A 100-mL Schlenk flask containing $PtCl_2$ (49 mg, 0.18 mmol) was dried under vacuum before it was charged with 2',5'-bis(5-bromo-2-ethynylphenyl)-1,1':4',1''-terphenyl (**11**) (400 mg, 649 µmol) and degassed toluene (60 mL). The mixture was further degassed by bubbling with argon for 15 min and then heated at 120 °C for 24 h. After completion of the reaction, the dark brown solution was cooled down to room temperature and the solvent was removed under reduced pressure. The residue was purified by silica gel column chromatography (eluent: *n*-hexane:dichloromethane = 4:1) to afford the title compound (84 mg, 21% yield) as yellow crystals, which was recrystallized for six times from methylene chloride/methanol (1:1, v/v) before use in the on-surface synthesis. ^{1}H NMR (250 MHz, Methylene Chloride-d_2) δ 7.73 – 7.61 (m, 10H), 7.60 (d, J = 2.0 Hz, 2H), 7.57 – 7.50 (m, 4H), 7.49 – 7.40 (m, 4H).; ^{13}C NMR (63 MHz, Methylene Chloride-d_2) δ 142.3, 138.1, 132.6, 132.3, 132.2, 131.7, 131.1, 130.1, 129.9, 129.6, 128.5, 127.6, 126.9, 126.5, 119.4; HRMS (MALDI-TOF): m/z Calcd for $C_{34}H_{20}Br_2$: 585.9932 $[M]^+$, found: 585.9931.

X-ray crystallographic analysis of monomer 1

Single crystals of 2,9-dibromo-7,14-diphenylbenzo[*k*]tetraphene (**1**) suitable for X-ray diffraction analysis were obtained by slow diffusion of methanol to its solution in dichloromethane as light yellow needles. The structure was deposited at Cambridge Crystallographic Data Centre (CCDC number: 2081474).

Crystal data for 2,9-dibromo-7,14-diphenylbenzo[*k*]tetraphene (**1**)

formula	$C_{34}H_{20}Br_2$		
molecular weight	588.32 gmol^{-1}		
absorption	μ = 3.338 mm^{-1} corrected with 6 crystal faces		
transmission	T_{min} = 0.4776, T_{max} = 0.8778		
crystal size	0.03 x 0.06 x 1.30 mm^3 light yellow needle		
space group	P 2_1/c (monoclinic)		
lattice parameters	a = 14.2762(10)Å		
(calculate from	b = 14.9361(15)Å	ß = 96.553(6)°	
7291 reflections with	c = 5.7716(5)Å		
2.7° < θ < 28.2°)	V = 1222.64(18)Å^3	z = 2	F(000) = 588
temperature	-80°C		
density	d_{xray} = 1.598 gcm^{-3}		

data collection

diffractometer	STOE IPDS 2T
radiation	Mo-K_α Graphitmonochromator
Scan – type	ω scans
Scan – width	1°
scan range	2° ≤ θ < 28°

	$-18 \leq h \leq 18$ $-17 \leq k \leq 19$ $-7 \leq l \leq 7$
number of reflections:	
measured	6131
unique	2984 (R_{int} = 0.0307)
observed	0.0642 ($\|F\|/\sigma(F) > 4.0$)

data correction, structure solution and refinement

corrections	Lorentz and polarisation correction.
Structure solution	Program: SIR-2004 (Direct methods)
refinement	Program: SHELXL-2017 (full matrix). 163 refined parameters, weighting scheme: $w=1/[\sigma^2(F_o^2) + (0.0163*P)^2+5.3*P]$ with $(Max(F_o^2,0)+2*F_c^2)/3$. H-atoms at calculated positions and refined with isotropic displacement parameters, non H- atoms refined anisotropically.
R-values	wR2 = 0.1326 (R1 = 0.0642 for observed reflections, 0.0985 for all reflections)
goodness of fit	S = 1.220
maximum deviation of parameters	0.001 * e.s.d
maximum peak height in diff. Fourier synthesis	0.5, -0.51 $eÅ^{-3}$
remark	molecule has Ci symmetry

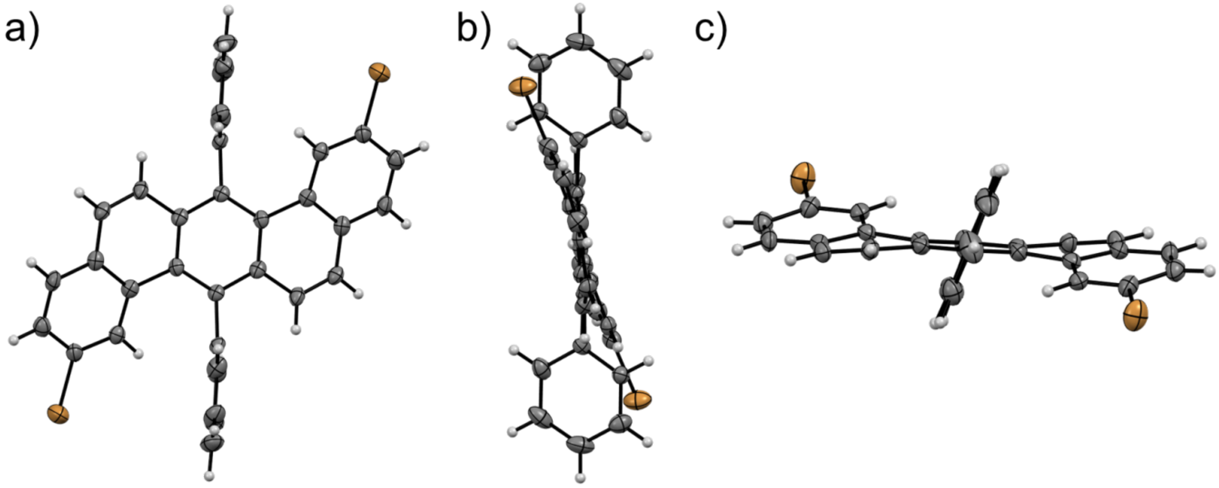

Figure S19 X-ray single-crystal structure of 2,9-dibromo-7,14-diphenylbenzo[*k*]tetraphene (**1**) with thermal ellipsoids set at 50% probability level: a) front view; b) side view; c) top view.

NMR spectra

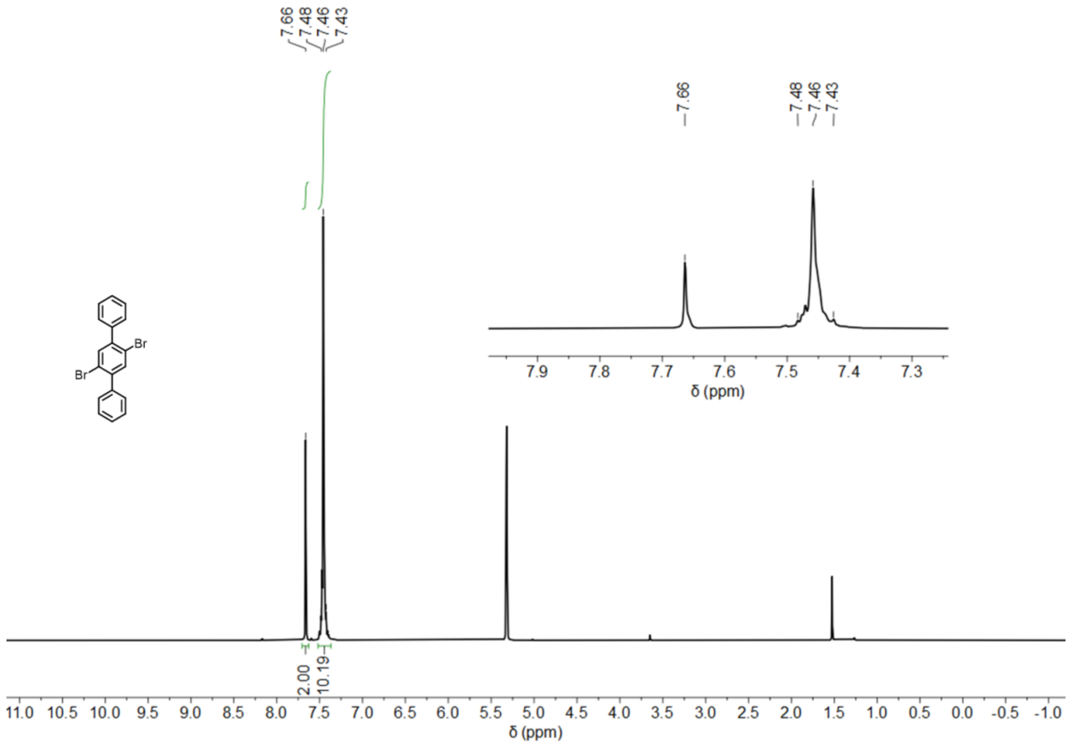

Figure S20 ^{1}H NMR spectrum of 1,4-dibromo-2,5-diphenylbenzene (**3**) in CD_2Cl_2 (300 MHz, 298 K).

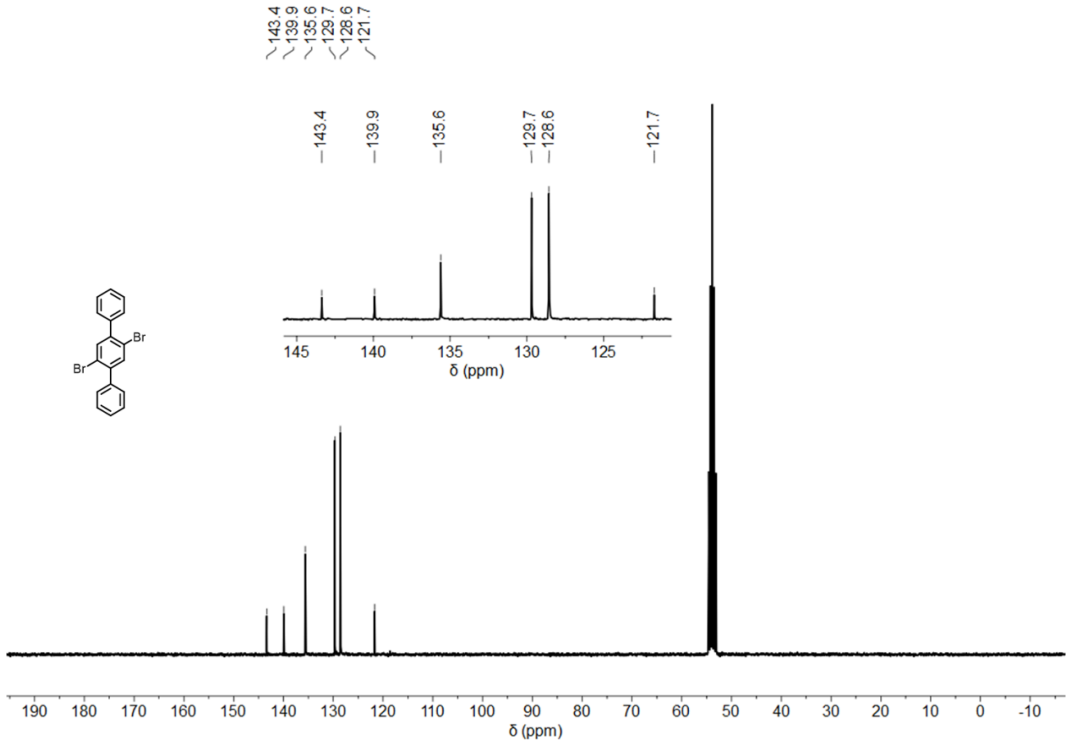

Figure S21 ^{13}C NMR spectrum of 1,4-dibromo-2,5-diphenylbenzene (**3**) in CD_2Cl_2 (75 MHz, 298 K).

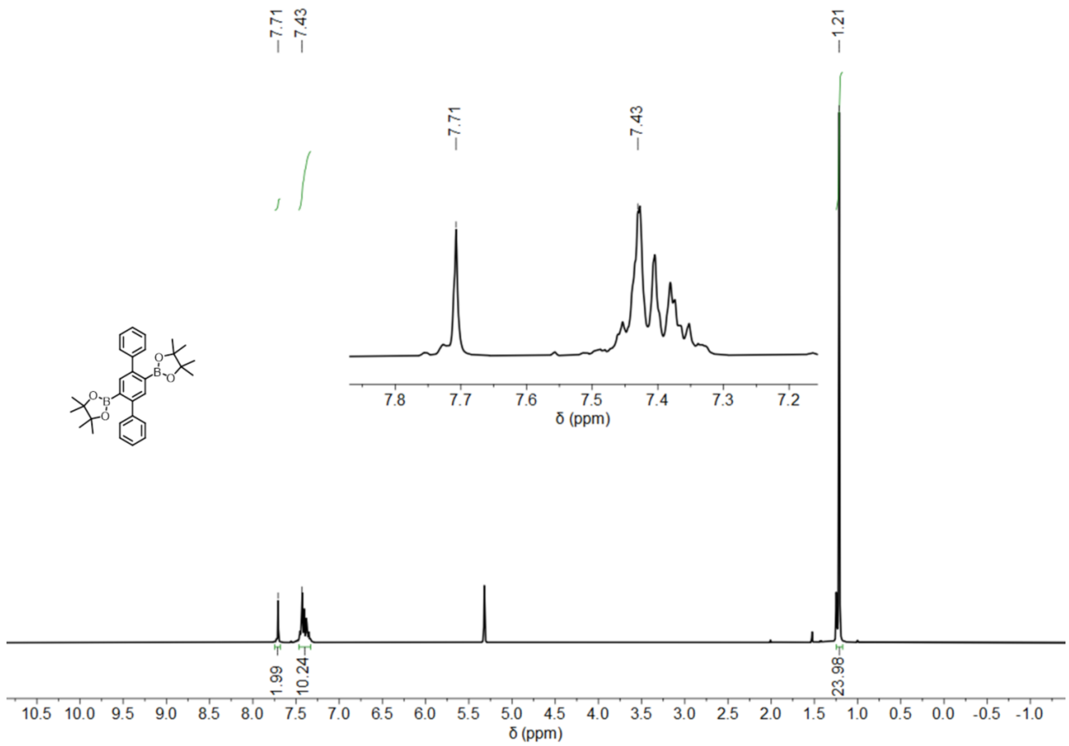

Figure S22 ^{1}H NMR spectrum of 1,4-bis(4,4,5,5-tetramethyl-1,3,2-dioxaborolan-2-yl)-2,5-diphenylbenzene (**4**) in CD_2Cl_2 (300 MHz, 298 K).

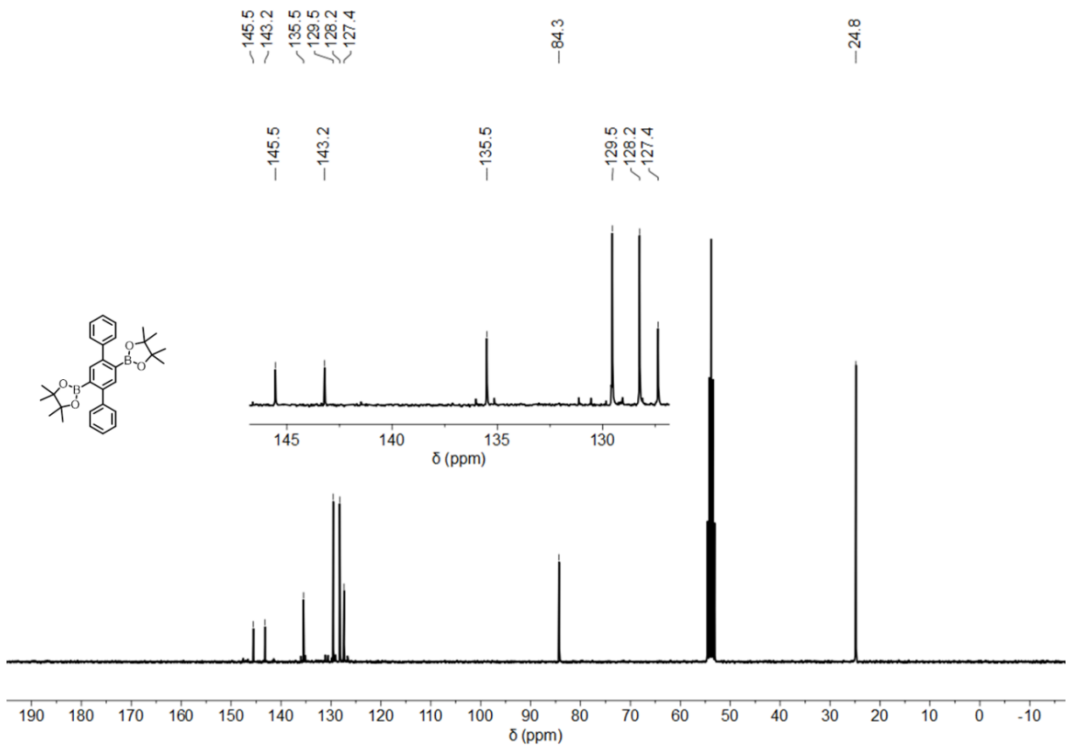

Figure S23 ^{13}C NMR spectrum of 1,4-bis(4,4,5,5-tetramethyl-1,3,2-dioxaborolan-2-yl)-2,5-diphenylbenzene (**4**) in CD_2Cl_2 (300 MHz, 298 K).

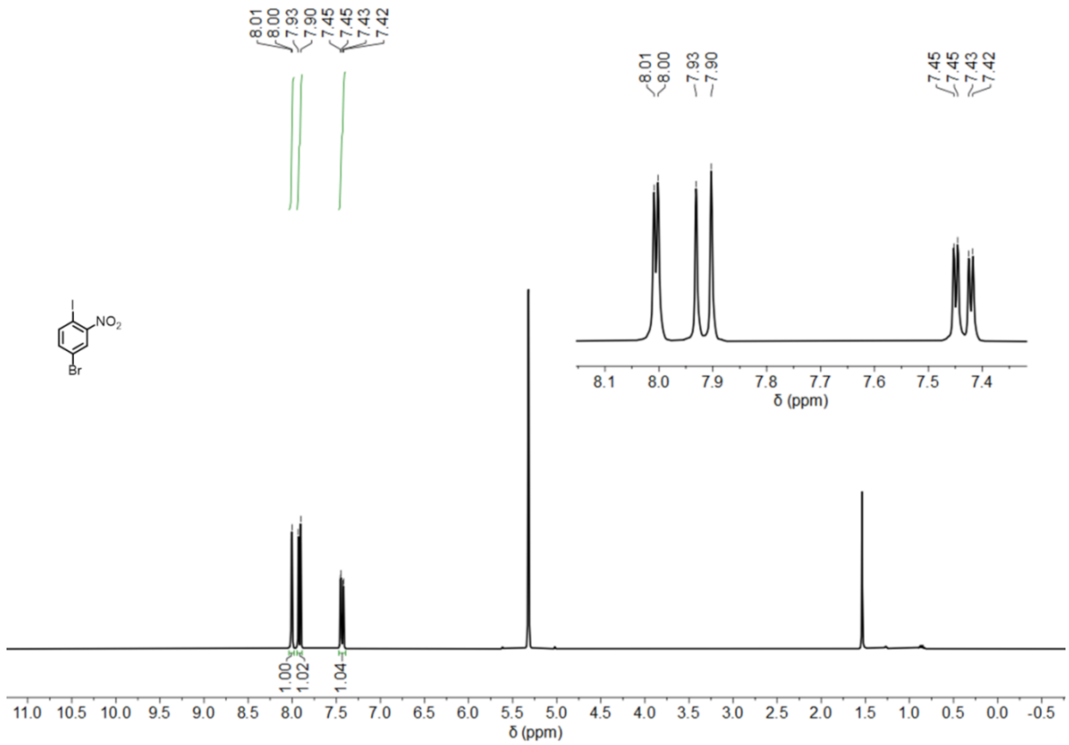

Figure S24 ^{1}H NMR spectrum of 4-bromo-1-iodo-2-nitrobenzene (**6**) in CD_2Cl_2 (300 MHz, 298 K).

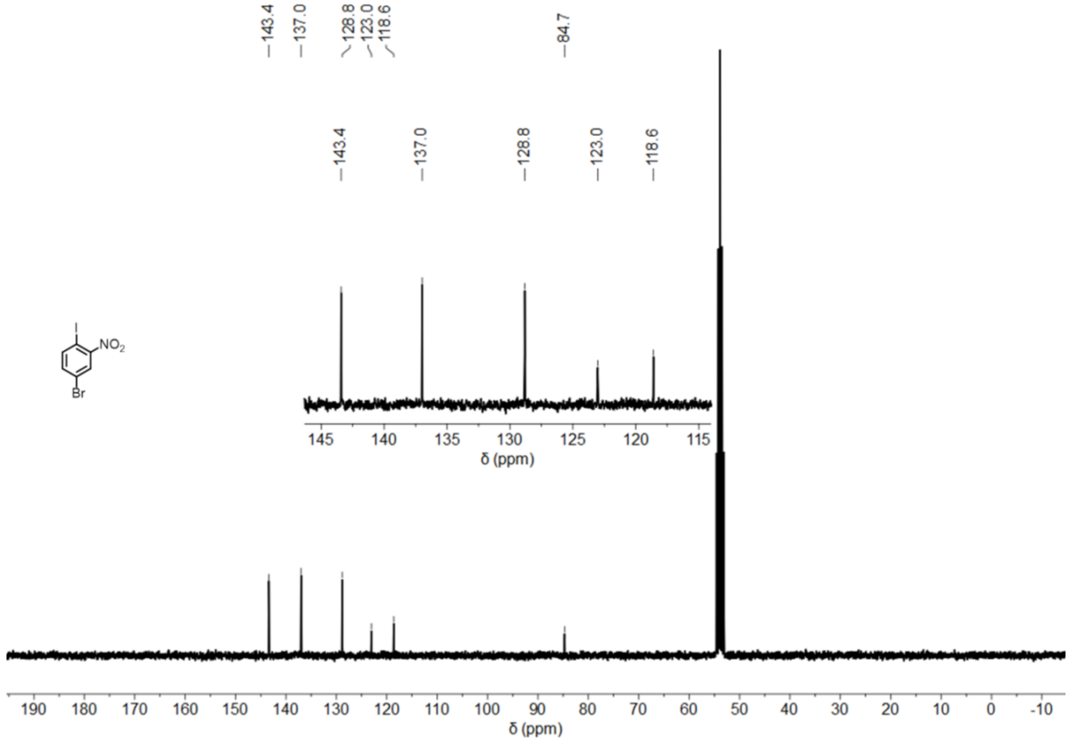

Figure S25 ^{13}C NMR spectrum of 4-bromo-1-iodo-2-nitrobenzene (**6**) in CD_2Cl_2 (75 MHz, 298 K).

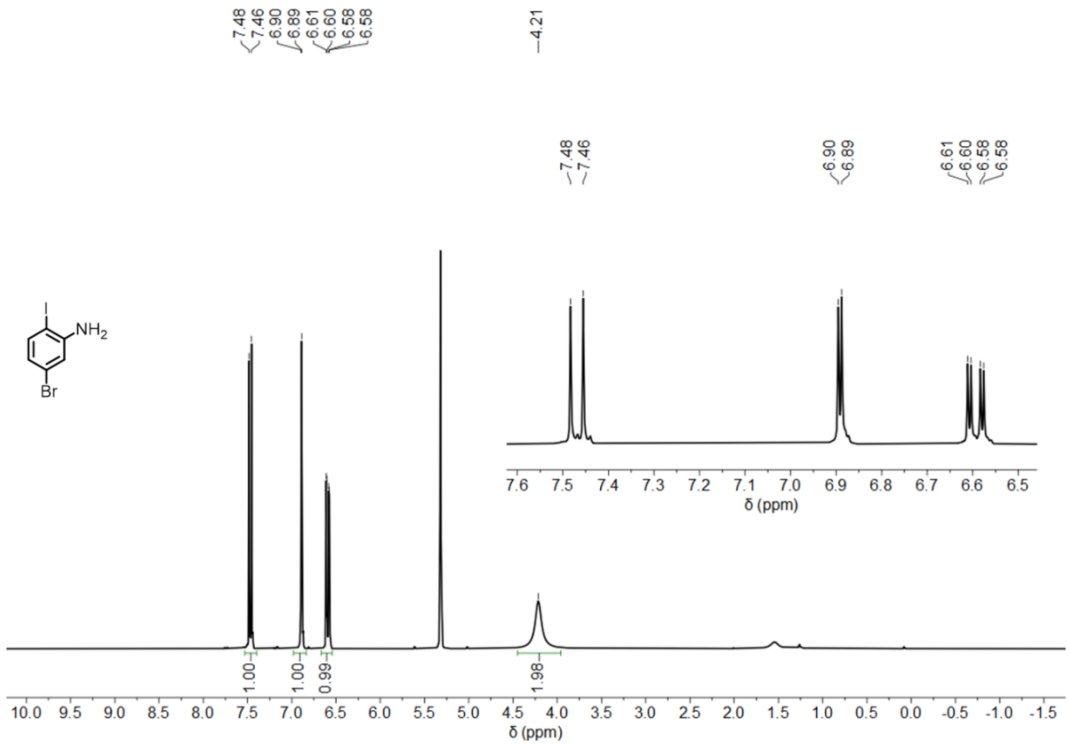

Figure S26 ^{1}H NMR spectrum of of 5-bromo-2-iodoaniline (**7**) in CD_2Cl_2 (300 MHz, 298 K).

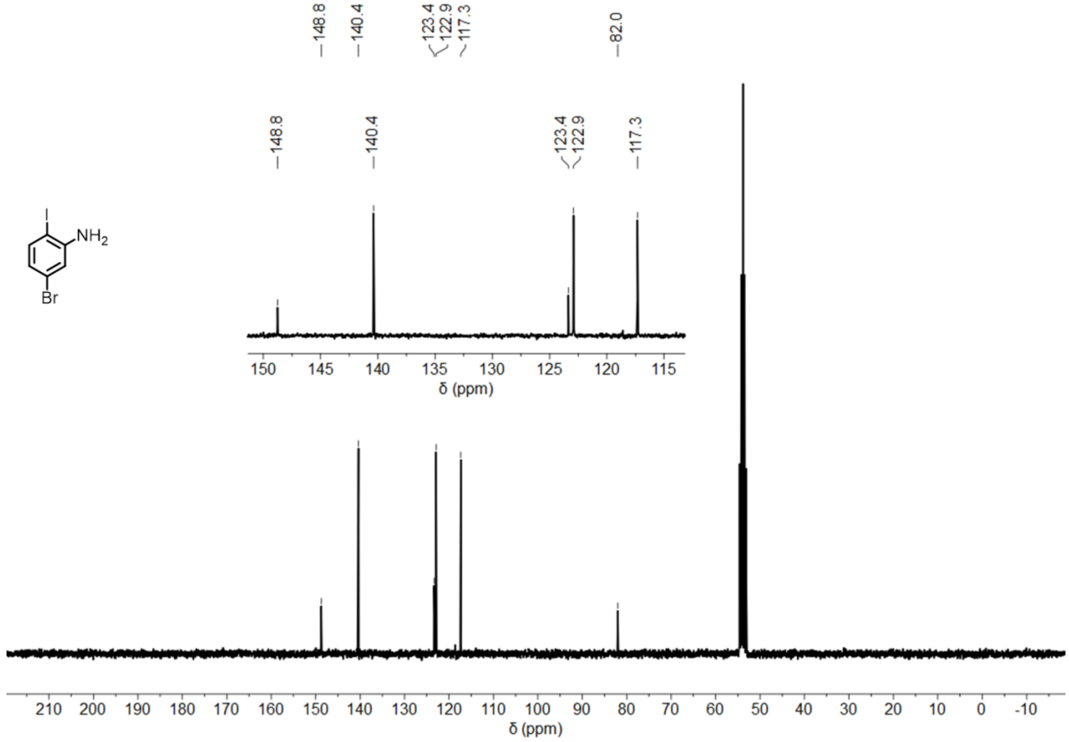

Figure S27 ^{13}C NMR spectrum of 5-bromo-2-iodoaniline (**7**) in CD_2Cl_2 (75 MHz, 298 K).

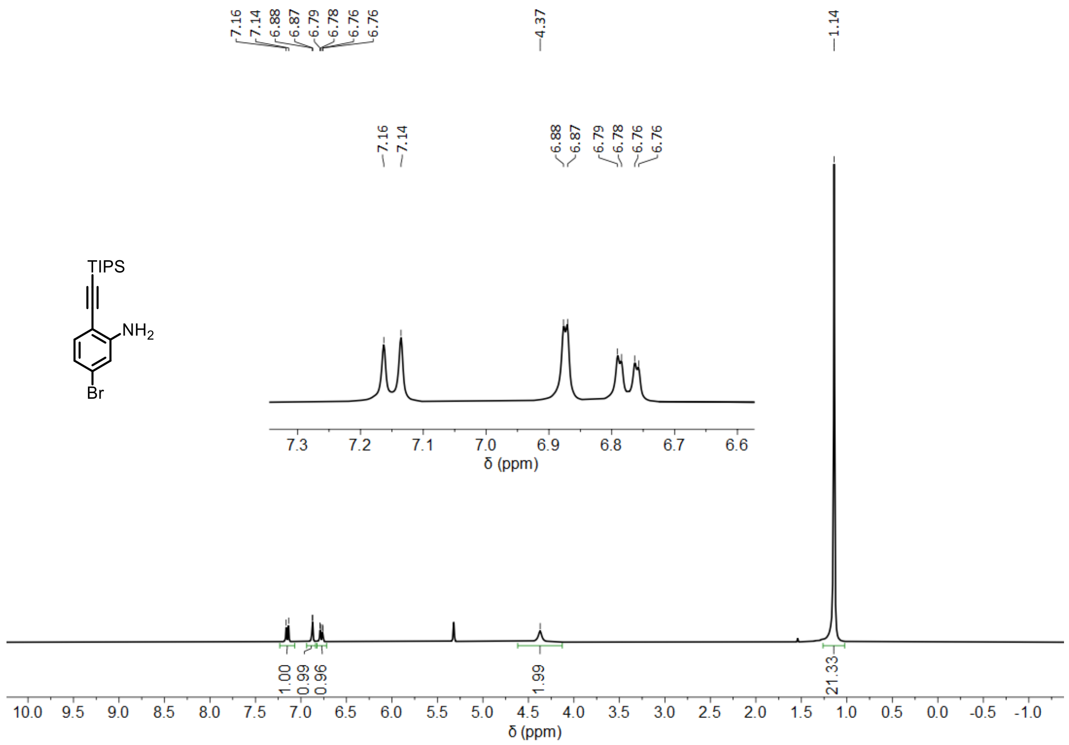

Figure S28 ^{1}H NMR spectrum of 5-bromo-2-((triisopropylsilyl)ethynyl)aniline (**8**) in CD_2Cl_2 (300 MHz, 298 K).

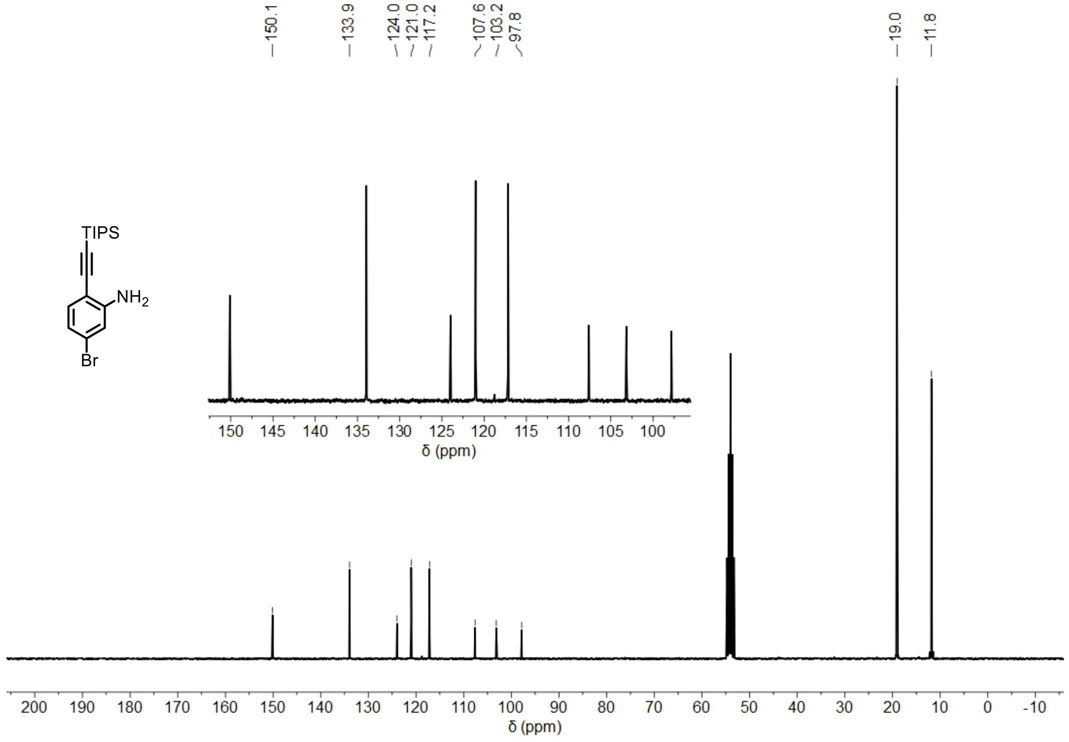

Figure S29 ^{13}C NMR spectrum of 5-bromo-2-((triisopropylsilyl)ethynyl)aniline (**8**) in CD_2Cl_2 (75 MHz, 298 K).

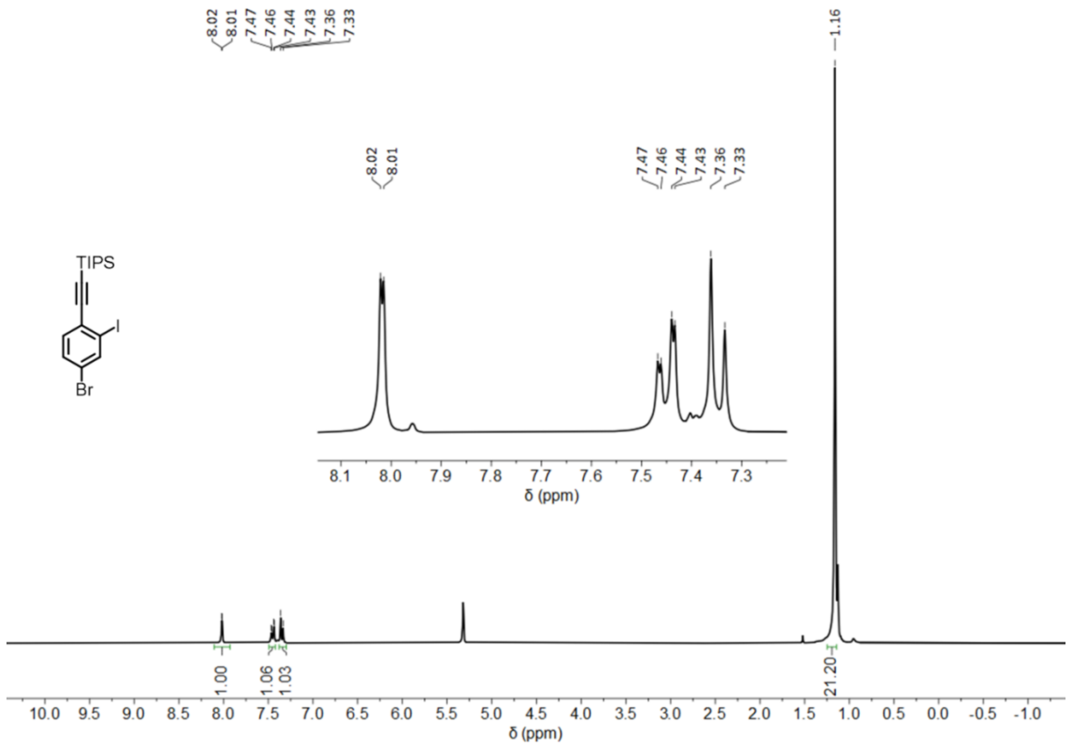

Figure S30 ^{1}H NMR spectrum of 4-bromo-2-iodo-1-((triisopropylsilyl)ethynyl)benzene (**9**) in CD_2Cl_2 (300 MHz, 298 K).

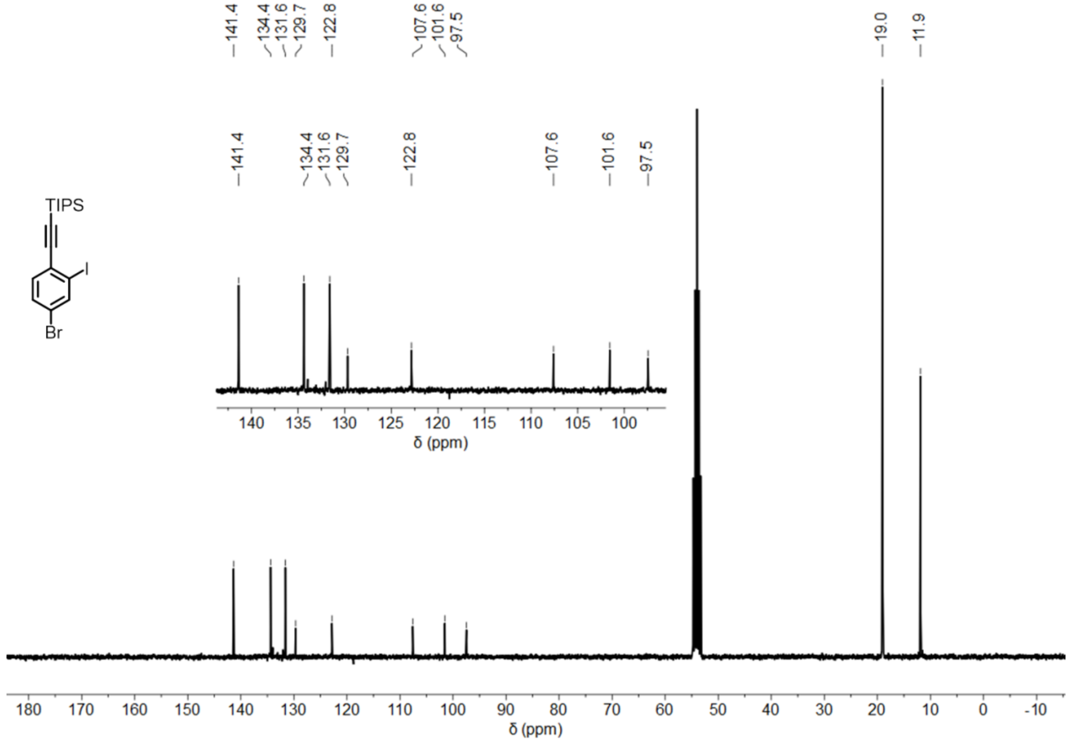

Figure S31 ^{13}C NMR spectrum of of 4-bromo-2-iodo-1-((triisopropylsilyl)ethynyl)benzene (**9**) in CD_2Cl_2 (75 MHz, 298 K).

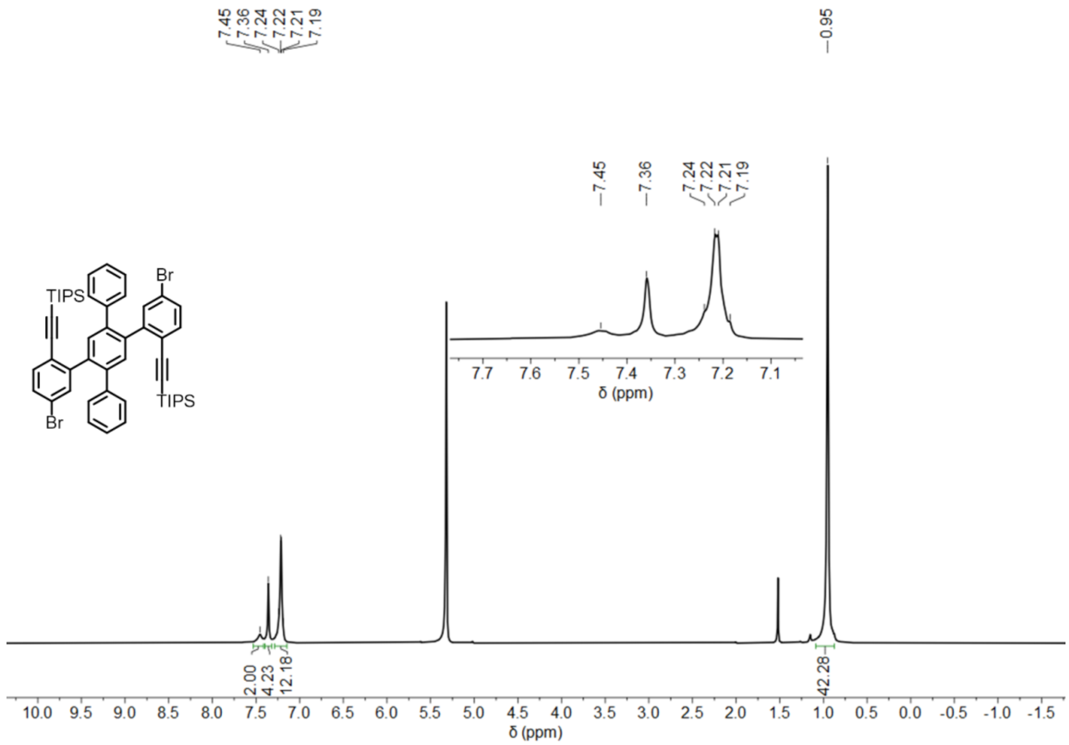

Figure S32 ^{1}H NMR spectrum of 2',5'-bis(5-bromo-2-((triisopropylsilyl)ethynyl)phenyl)-1,1':4',1''-terphenyle (**10**) in CD_2Cl_2 (300 MHz, 298 K).

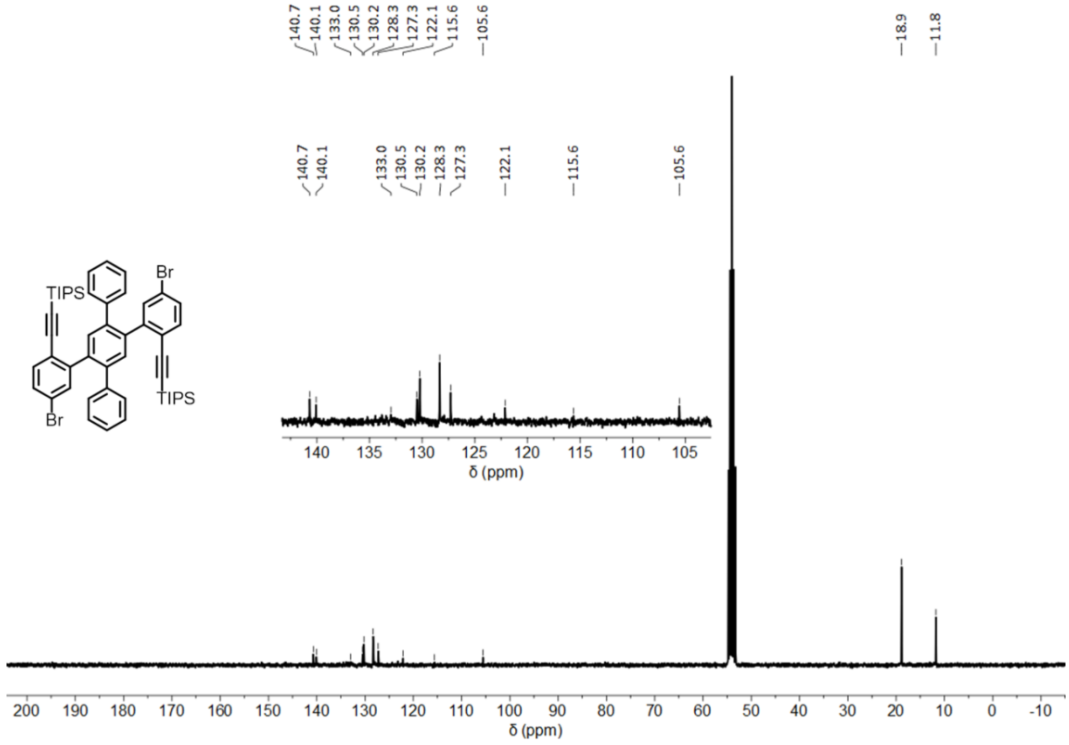

Figure S33 ^{13}C NMR spectrum of 2',5'-bis(5-bromo-2-((triisopropylsilyl)ethynyl)phenyl)-1,1':4',1''-terphenyle (**10**) in CD_2Cl_2 (300 MHz, 298 K).

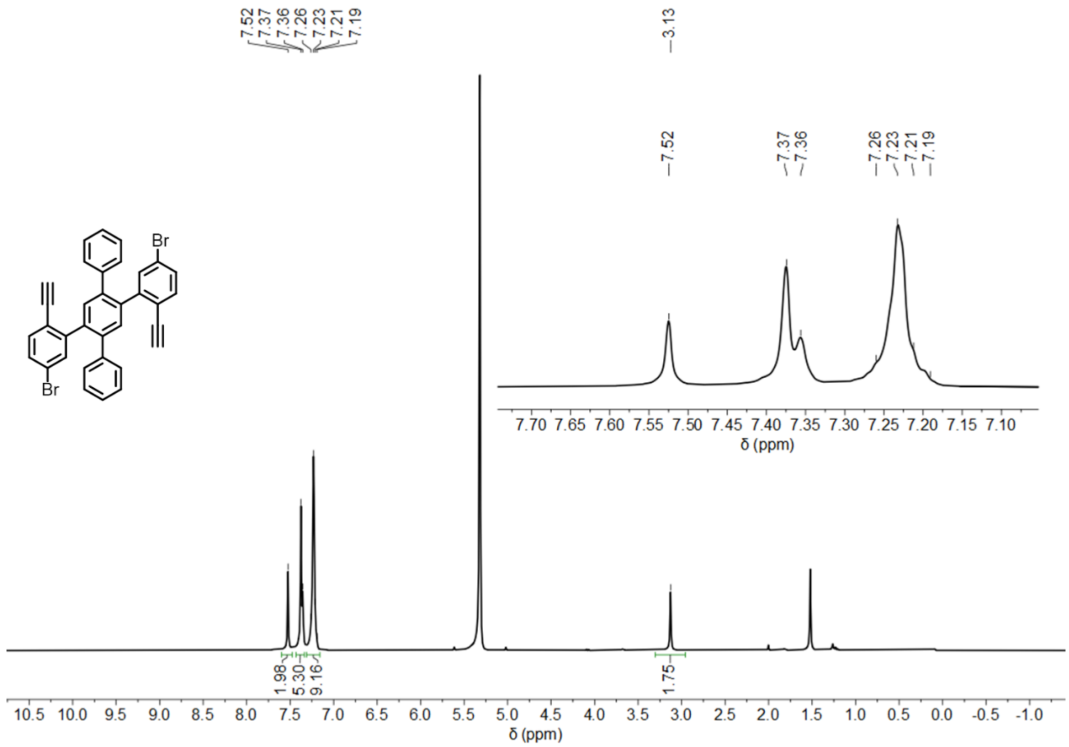

Figure S34 ^{1}H NMR spectrum of 2',5'-bis(5-bromo-2-ethynylphenyl)-1,1':4',1''-terphenyl (**11**) in CD_2Cl_2 (300 MHz, 298 K).

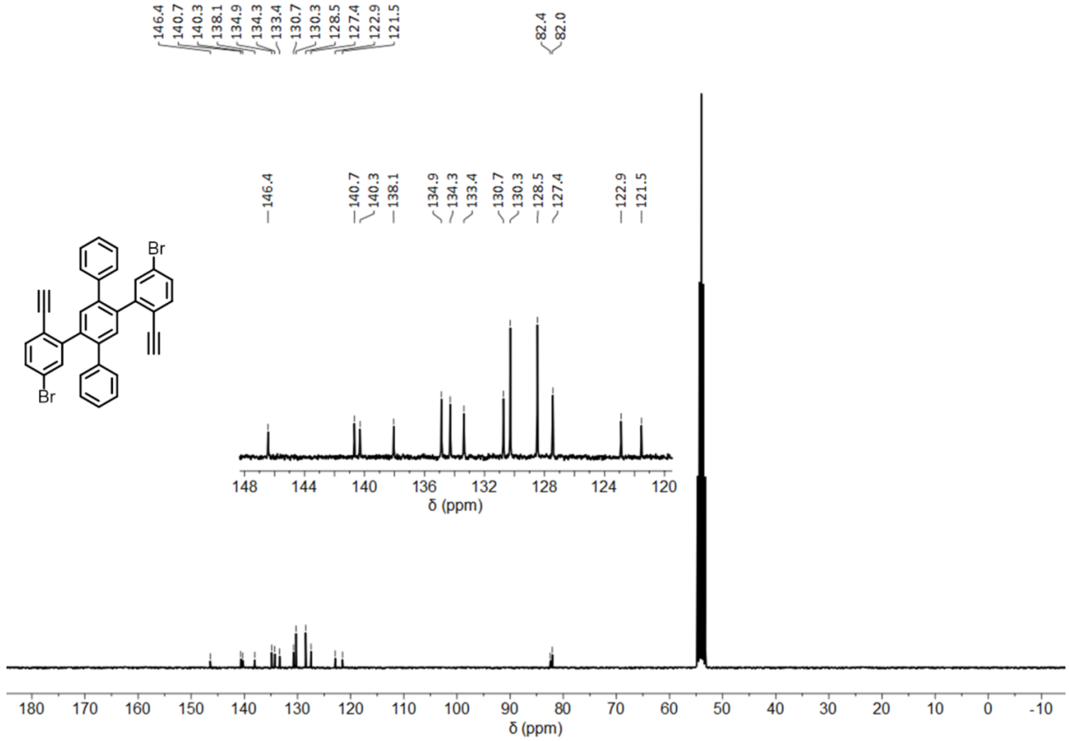

Figure S35 ^{13}C NMR spectrum of 2',5'-bis(5-bromo-2-ethynylphenyl)-1,1':4',1''-terphenyl (**11**) in CD_2Cl_2 (300 MHz, 298 K).

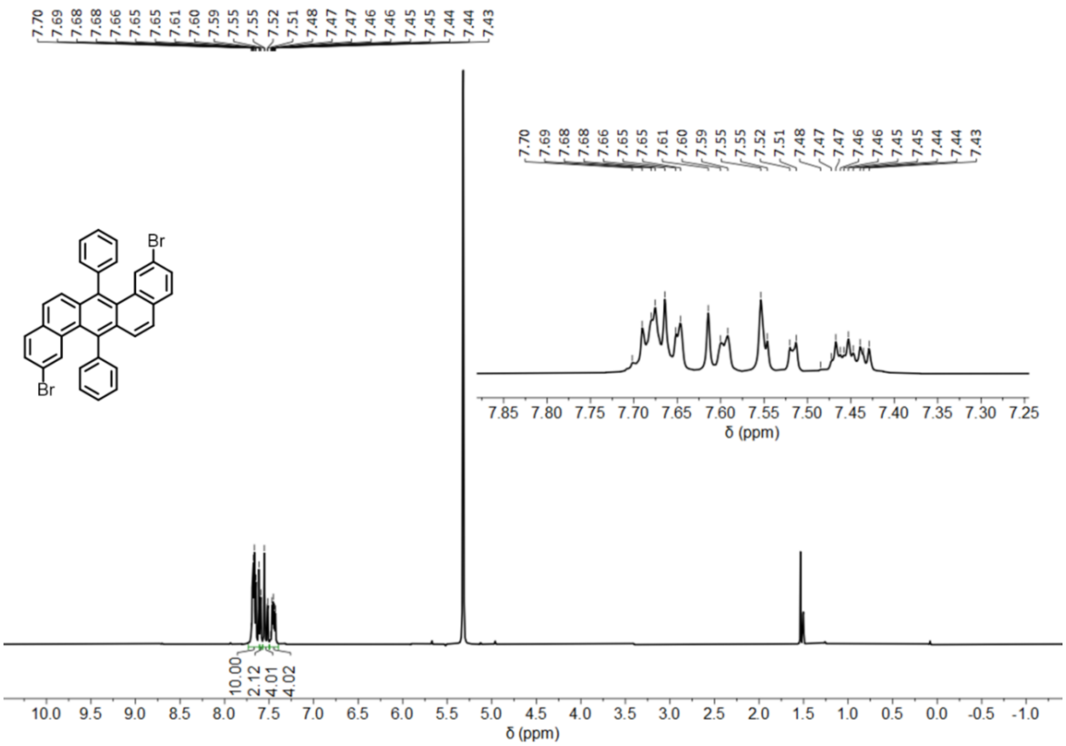

Figure S36 ^{1}H NMR spectrum of 2,9-dibromo-7,14-diphenylbenzo[*k*]tetraphene (**1**) in CD_2Cl_2 (250 MHz, 298 K).

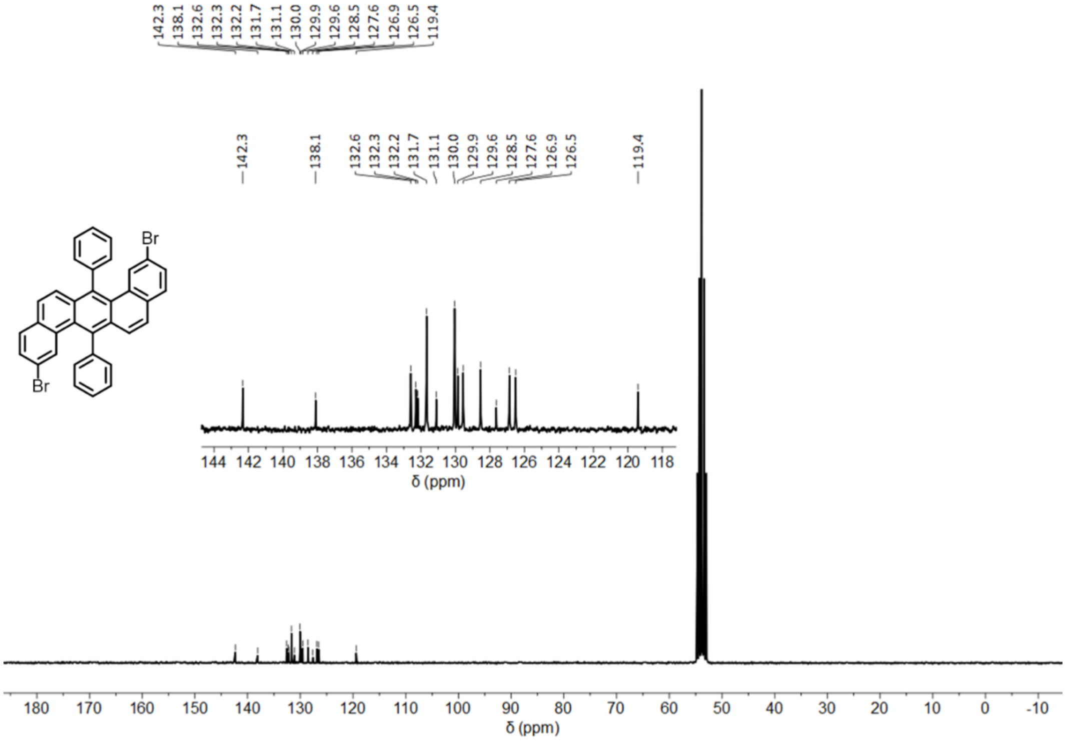

Figure S37 ^{13}C NMR spectrum of 2,9-dibromo-7,14-diphenylbenzo[*k*]tetraphene (**1**) in CD_2Cl_2 (63 MHz, 298 K).

References

1. Gross, L. *et al.* The chemical structure of a molecules resolved by atomic force microscopy. *Science* **325**, 1110–1114 (2009).
2. Kresse, G. & Furthmüller, J. Efficient iterative schemes for ab initio total-energy calculations using a plane-wave basis set. *Phys. Rev. B* **5**, 11169–11186 (1996).
3. Blöchl, P. E. Projector augmented-wave method. *Phys. Rev. B* **50**, 17953–17979 (1994).
4. Kresse, G. & Joubert, D. From ultrasoft pseudopotentials to the projector augmented-wave method. *Phys. Rev. B* **59**, 1758–1775 (1999).
5. Hamada, I. Van der Waals density functional made accurate. *Phys. Rev. B* **89**, 121103(R)-121107(R) (2014).
6. Björk, J. & Stafström, S. Adsorption of Large Hydrocarbons on Coinage Metals: A van der Waals Density Functional Study. *ChemPhysChem* **15**, 2851–2858 (2014).
7. Henkelman, G., Uberuaga, B. P. & Jónsson, H. Climbing image nudged elastic band method for finding saddle points and minimum energy paths. *J. Chem. Phys.* **113**, 9901–9904 (2000).
8. Kästner, J. & Sherwood, P. Superlinearly converging dimer method for transition state search. *J. Chem. Phys.* **128**, 014106–014111 (2008).
9. Chase, M. W. NIST-JANAF Themochemical Tables, Fourth Edition. *J. Phys. Chem. Ref. Data* **9**, 1310 (1998).
10. Campbell, C. T., Sprowl, L. H. & Árnadóttir, L. Equilibrium Constants and Rate Constants for Adsorbates: Two-Dimensional (2D) Ideal Gas, 2D Ideal Lattice Gas, and Ideal Hindered Translator Models. *J. Phys. Chem. C* **120**, 10283–10297 (2016).
11. Hutter, J., Iannuzzi, M., Schiffmann, F. & Vandevondele, J. Cp2k: Atomistic simulations of condensed matter systems. *WIREs Comput. Mol. Sci.* **4**, 15–25 (2014).
12. Hapala, P. *et al.* Mapping the electrostatic force field of single molecules from high-resolution scanning probe images. *Nat. Commun.* **7**, 11560 (2016).
13. Yakutovich, A. V. *et al.* AiiDAlab – an ecosystem for developing, executing, and sharing

scientific workflows. *Comput. Mater. Sci.* **188**, 110165 (2021).

14. VandeVondele, J. & Hutter, J. Gaussian basis sets for accurate calculations on molecular systems in gas and condensed phases. *J. Chem. Phys.* **127**, 114105–114113 (2007).

15. Goedecker, S. & Teter, M. Separable dual-space Gaussian pseudopotentials. *Phys. Rev. B* **54**, 1703–1710 (1996).

16. Perdew, J. P., Burke, K. & Ernzerhof, M. Generalized gradient approximation made simple. *Phys. Rev. Lett.* **77**, 3865–3868 (1996).

17. Grimme, S., Antony, J., Ehrlich, S. & Krieg, H. A consistent and accurate ab initio parametrization of density functional dispersion correction (DFT-D) for the 94 elements H-Pu. *J. Chem. Phys.* **132**, 154104–154122 (2010).

18. Wei, B., Zhang, D., Chen, Y. H., Lei, A. & Knochel, P. Preparation of Polyfunctional Biaryl Derivatives by Cyclolanthanation of 2-Bromobiaryls and Heterocyclic Analogues Using nBu2LaCl·4 LiCl. *Angew. Chem. Int. Ed.* **58**, 15631–15635 (2019).

19. Cho, I. *et al.* Design, Synthesis, and Versatile Processing of Indolo[3,2-b]indole-Based π-Conjugated Molecules for High-Performance Organic Field-Effect Transistors. *Adv. Funct. Mater.* **26**, 2966–2973 (2016).

20. Kobayashi, S., Hirano, T., Iwakiri, K., Miyamoto, H. & Atsuo Nakazaki. Total Synthesis of (−)-Flustramine B via One-Pot Intramolecular Ullmann Coupling and Claisen Rearrangement. *Heterocycles* **79**, 805–820 (2009).
